# Chloroplast genomes of *Arabidopsis halleri* ssp. *gemmifera* and *Arabidopsis lyrata* ssp. *petraea:* Structures and comparative analysis

**DOI:** 10.1038/s41598-017-07891-5

**Published:** 2017-08-08

**Authors:** Sajjad Asaf, Abdul Latif Khan, Muhammad Aaqil Khan, Muhammad Waqas, Sang-Mo Kang, Byung-Wook Yun, In-Jung Lee

**Affiliations:** 1 School of Applied Biosciences, Kyungpook National University, Daegu, 41566 Republic of Korea; 2 Chair of Oman’s Medicinal Plants & Marine Natural Products, University of Nizwa, Nizwa, 616 Oman

## Abstract

We investigated the complete chloroplast (cp) genomes of non-model *Arabidopsis halleri* ssp. *gemmifera* and *Arabidopsis lyrata* ssp. *petraea* using Illumina paired-end sequencing to understand their genetic organization and structure. Detailed bioinformatics analysis revealed genome sizes of both subspecies ranging between 154.4~154.5 kbp, with a large single-copy region (84,197~84,158 bp), a small single-copy region (17,738~17,813 bp) and pair of inverted repeats (IRa/IRb; 26,264~26,259 bp). Both cp genomes encode 130 genes, including 85 protein-coding genes, eight ribosomal RNA genes and 37 transfer RNA genes. Whole cp genome comparison of *A*. *halleri* ssp. *gemmifera* and *A*. *lyrata* ssp. *petraea*, along with ten other *Arabidopsis* species, showed an overall high degree of sequence similarity, with divergence among some intergenic spacers. The location and distribution of repeat sequences were determined, and sequence divergences of shared genes were calculated among related species. Comparative phylogenetic analysis of the entire genomic data set and 70 shared genes between both cp genomes confirmed the previous phylogeny and generated phylogenetic trees with the same topologies. The sister species of *A*. *halleri* ssp. *gemmifera is A*. *umezawana*, whereas the closest relative of *A*. *lyrata spp*. *petraea* is *A*. *arenicola*.

## Introduction

The chloroplast is an active metabolic centre that paves the way for sustaining plant growth and development by converting solar energy to carbohydrates through the process of photosynthesis^1, 2^. It serves as a metabolic epicentre in cellular metabolic reactions^2^. The chloroplast (cp) genome is composed of genetic information that encodes synthesis of many key proteins involved in photosynthesis and metabolomic processes^1, 2^. In addition, the cp genome contains valuable information for plant systematics, as it is inherited maternally in most angiosperms^3, 4^. Substitution rates in plant cp genomes are much lower than those in nuclear genomes^5^. Plant cp genomes are also valuable sources of genetic markers for phylogenetic analyses because of their very low levels of recombination^6, 7^.

The advent of high-throughput sequencing technologies has facilitated rapid progress in the field of chloroplast genomics^7, 8^. While the first complete nucleotide sequences of *Nicotiana tabacum* and *Marchantia polymorpha* were painstakingly generated using clone sequencing of plasmid and cosmid libraries^9, 10^, there are now over 800 cp genomes (including 300 from crops and trees) that have been sequenced and deposited in the NCBI Organelle Genome Resources database. The evolution of cp genomes in terrestrial plants may now be studied using these database resources^11^. Chloroplast genome sequences have shown significant variation within and between plant species in terms of both sequence and structural variations^12^. Such information in chloroplast genomes is essential for understanding the climatic adaptation of economically important crops, for the selection of alternatives to breeding closely related species and for the identification and conservation of valuable traits^8, 13^.

Previous studies have suggested that cp genomes sequences increase phylogenetic resolution at lower taxonomic levels and that they are effective tools for plant phylogenetic and population genetic analyses^6, 8, 14^. The typical chloroplast genome in angiosperms has a conserved quadripartite structure, with one large single-copy (LSC) region, one small single-copy (SSC) region, and two copies of an inverted repeat (IR)^15^. Despite its conservation of structure, the size of cp genomes fluctuates between species, ranging from 107 kb (*Cathaya argyrophylla*) to 218 kb (*Pelargonium hortorum*)^11, 16, 17^. The current rapid development of DNA sequencing technology has resulted in the extensive use of cp genomes as molecular markers in numerous molecular phylogenetic studies.

*Arabidopsis thaliana* is a well-known model organism because of its rapid life cycle, small nuclear genome, amenability to genetic analysis and practical use in breeding biology^18–20^. The steadily increasing knowledge of *Arabidopsis* has benefited a new era of functional genomics and evolutionary analyses focused on several taxa in *Brassicaceae* with varying divergence from the model species^21, 22^. In general, three major lineages have been recognized, namely, *A*. *arenosa*, *A*. *halleri* and *A*. *lyrata* ^23^, and most species or subspecies can be categorized within these three lineages.

*A*. *lyrata* ssp. *petraea* (2*n* = 16/32) is a perennial herb that diverged from a common ancestor with *A*. *thaliana* approximately 10 million years ago^24^. Despite its evolutionary proximity, its nuclear genome size is estimated to be between 230 to 245 Mb, or one and a half times larger than that of the *A*. *thaliana* ^25, 26^. *A*. *halleri* ssp. *gemmifera* (2*n* = 16) is a stoloniferous diploid perennial herb with a disjunctive distribution in eastern Asia, including Japan and Taiwan, and its nuclear genome is 40–60% larger than that of *A*. *thaliana* ^25–28^. In contrast to *A*. *lyrata*, the geographical distribution of *A*. *halleri* rarely extends into northern latitudes, and the species is more tolerant of competition. *A*. *halleri* grows on acidic, neutral and oligotrophic soils, as well as on soils with high heavy metal contents^28, 29^. *A*. *halleri* can accumulate zinc and cadmium in its aerial parts. On the other hand, *A*. *lyrata* is sensitive to metals, suggesting that metal hyperaccumulation in *A*. *halleri* is mainly derived from its ecological niche. Moreover, the available data suggest that plants growing in contaminated metallic and non-metallic soils could share such traits^30^.

The genus *Arabidopsis* is frequently affected by the processes of hybridization and introgression^31^, as both *A*. *lyrata* and *A*. *halleri* possess diploid structures that are reported hybrids from *A*. *kamchatica* on the basis of either nuclear genes or self-incompatibility alleles^31–34^. There has been clear evidence of admixture between tetraploid *A*. *lyrata* ssp. *petraea* and *A*. *arenosa* ssp. *Borbasii* ^34^, whereas *A*. *halleri* ssp. *gemmifera* is a close relative of *Arabidopsis thaliana* ^35^. Precise phylogenetic placement has proven difficult according to Al-Shehbaz and OKane (2002)^36^, Schmickl *et al*.^37^, Hohmann *et al*.^38^, and Novikova *et al*.^34^, and requires further investigation. *A*. *halleri* and *A*. *lyrata*, along with their subspecies, on the other hand, are extensively used as outgroups in comparative plant systematic studies^24, 39, 40^. However, there is little information available on their genetic structure, especially their chloroplast genomes or their detailed phylogenetic placement. The current study presents the cp genomes of *A*. *lyrata* ssp. *petraea* and *A*. *halleri* ssp. *gemmifera* for the first time. In this study, we sequenced the complete chloroplast genomes of *A*. *halleri* ssp. *gemmifera* (GenBank accession number: KU764767) and *A*. *lyrata* ssp. *petraea* (GenBank accession number: KU764768). We aimed to elucidate the global patterns of structural variations in the cp genomes of *A*. *halleri* ssp. *gemmifera* and *A*. *lyrata* ssp. *petraea* and compare them with cp genomes of other *Arabidopsis* species available from NCBI.

## Results

### Chloroplast genome features and the structure of two *Arabidopsis* subspecies

**Figure 1:**
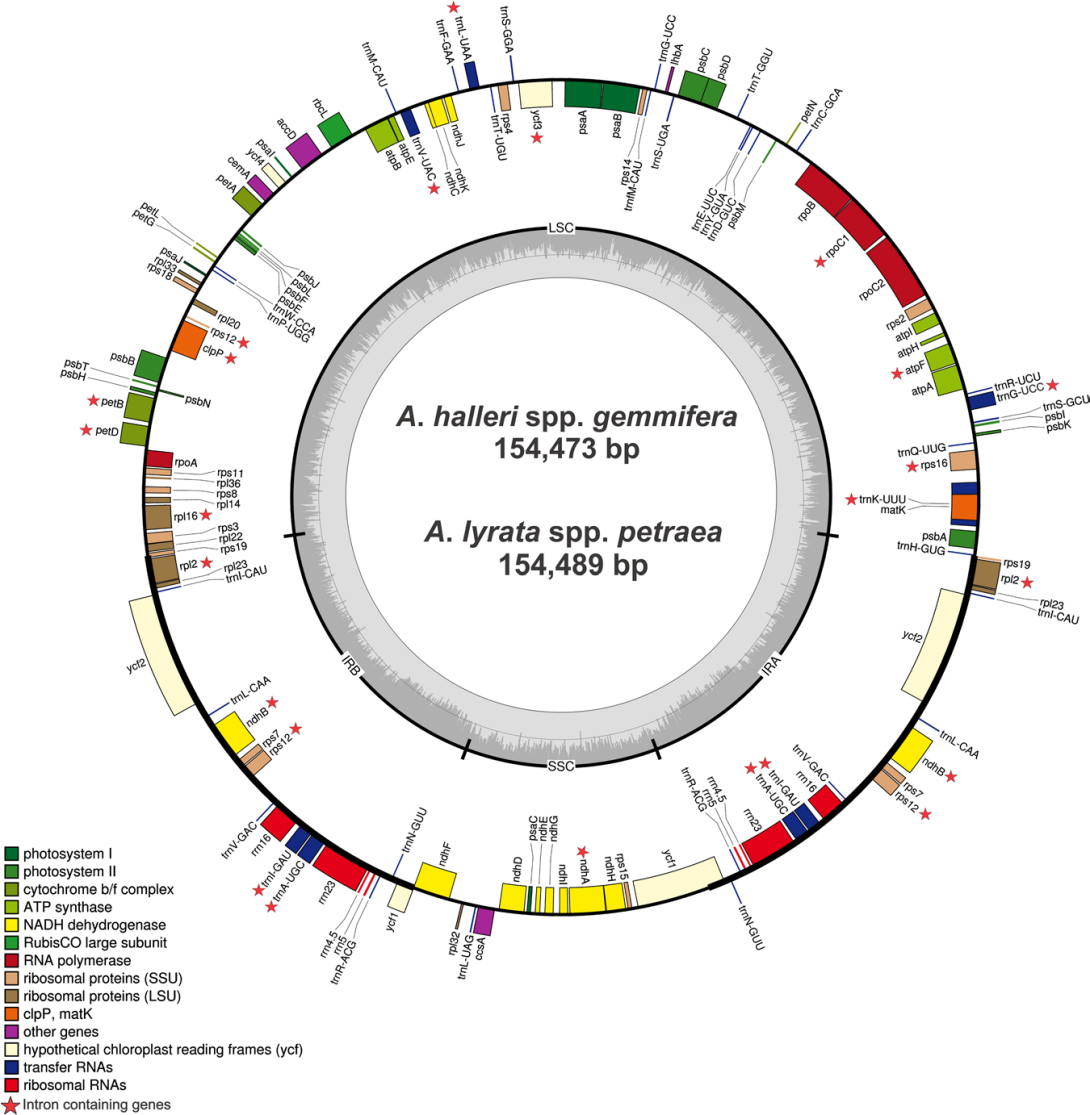
Gene map of the *A*. *halleri* ssp. *gemmifera* and *A*. *lyrata* ssp. *petraea* chloroplast genomes. Genes drawn inside the circle are transcribed clockwise, and those outside the circle are transcribed counter clockwise. The asterisks indicate intron-containing genes. Genes belonging to different functional groups are colour-coded. The darker grey in the inner circle corresponds to GC content, and the lighter grey corresponds to AT content.

Two *Arabidopsis* species, *A*. *halleri* ssp. *gemmifera* and *A*. *lyrata* ssp. *petraea*, were sequenced on an Illumina HiSeq. 2000 to produce 63,528,604 and 67,938,537 bp paired-end raw reads, respectively. The average read length was 101 bp, and the cp genomes of *A*. *halleri* ssp. *gemmifera* and *A*. *lyrata* ssp. *petraea* received 2232.81x and 2173.5x coverage, respectively. The four junction regions for each resulting cp genome were also confirmed by PCR-based Sanger sequencing with four pairs of primers (Table S2). The cp genome sizes of *A*. *halleri* ssp. *gemmifera* and *A*. *lyrata* ssp. *petraea* were 154,473 and 154,489 bp, respectively (Table 1, Fig. 1). The genomes displayed a typical quadripartite structure, as shown by most angiosperms. This included a large single-copy region (LSC; 84197 bp and 84158 bp), a small single-copy region (SSC; 17738 bp and 17813 bp) and a pair of inverted repeats (IRa/IRb; 26264 bp and 26259 bp) in *A*. *halleri* ssp. *gemmifera* and *A*. *lyrata* ssp. *petraea*, respectively. The GC contents (36.4%) of both *A*. *halleri* ssp. *gemmifera* and *A*. *lyrata* ssp. *petraea* were very similar to those in the cp genomes of other *Arabidopsis* species (Table 1). However, the GC contents were unequally distributed in different fragments of the cp genomes, with the highest values in the IR regions (42.3%), median values in the LSC regions (34.1%) and the lowest values in the SSC regions (29.4%) (Table 1). The high GC content of the IR regions might be due to the presence of eight ribosomal RNA (rRNA) sequences in these regions.

**Table 1:** Summary of complete chloroplast genomes for twelve Arabidopsis species.

Region	*A. are*	*A. ceb*	*A. h. gem*	*A. l. pet*	*A. ped*	*A. tha*	*A. aren*	*A. cro*	*A. neg*	*A. pet*	*A. sue*	*A. ume*
**LSC**												
Length (bp)	84464	84160	84197	84158	84478	84170	84234	84336	84397	84478	84090	84251
GC (%)	34.2	34.1	34.1	34.1	34	34	34.1	34.1	34.2	34.2	34	34.1
Length (%)	54.53	54.47	54.50	54.47	54.53	54.48	54.4	54.5	54.5	54.5	54.4	54.4
**SSC**												
Length (bp)	17885	17830	17738	17813	17873	17780	17859	17862	17882	18875	17755	17872
GC (%)	29.4	29.4	29.4	29.4	29.5	29.3	29.4	29.4	29.4	29.4	29.4	29.4
Length (%)	11.54	11.54	11.48	11.53	11.53	11.50	11.5	11.54	11.55	12.1	11.5	11.55
**IR**												
Length (bp)	26261	26257	26264	26259	26272	26264	26258	26265	26260	26256	26259	26261
GC (%)	42.3	42.3	42.3	42.3	42.3	42.3	42.3	42.3	42.3	42.3	42.3	42.3
Length (%)	16.95	16.99	17.00	16.99	16.96	17.00	16.9	16.9	16.9	16.9	17	16.9
**Total**												
GC (%)	36.4	36.4	36.4	36.4	36.3	36.3	36.4	36.4	36.4	36.4	36.3	36.4
Length (%)	154871	154504	154473	154489	154895	154478	154610	154728	154799	154865	154366	154645

**A. are** = *A*. *arenosa*; **A. ceb** = *A*. *cebennensis*; **A. h. gem** = *A*. *halleri ssp*. *gemmifera*; **A. l. pet** = *A*. *lyrata* ssp. *petraea*; **A. ped** = *A*. *pedemontana*; **A. tha** = *A*. *thanliana*; **A. aren** = *A*. *arenicola*; **A. cro** = *A*. *croatica*; **A. neg** = *A*. *neglecta*; **A. pet** = *A*. *petrogenea*; **A. sue** = *A*. *suecia*; **A. ume** = *A*. *umezawana*.

**Figure 2:**
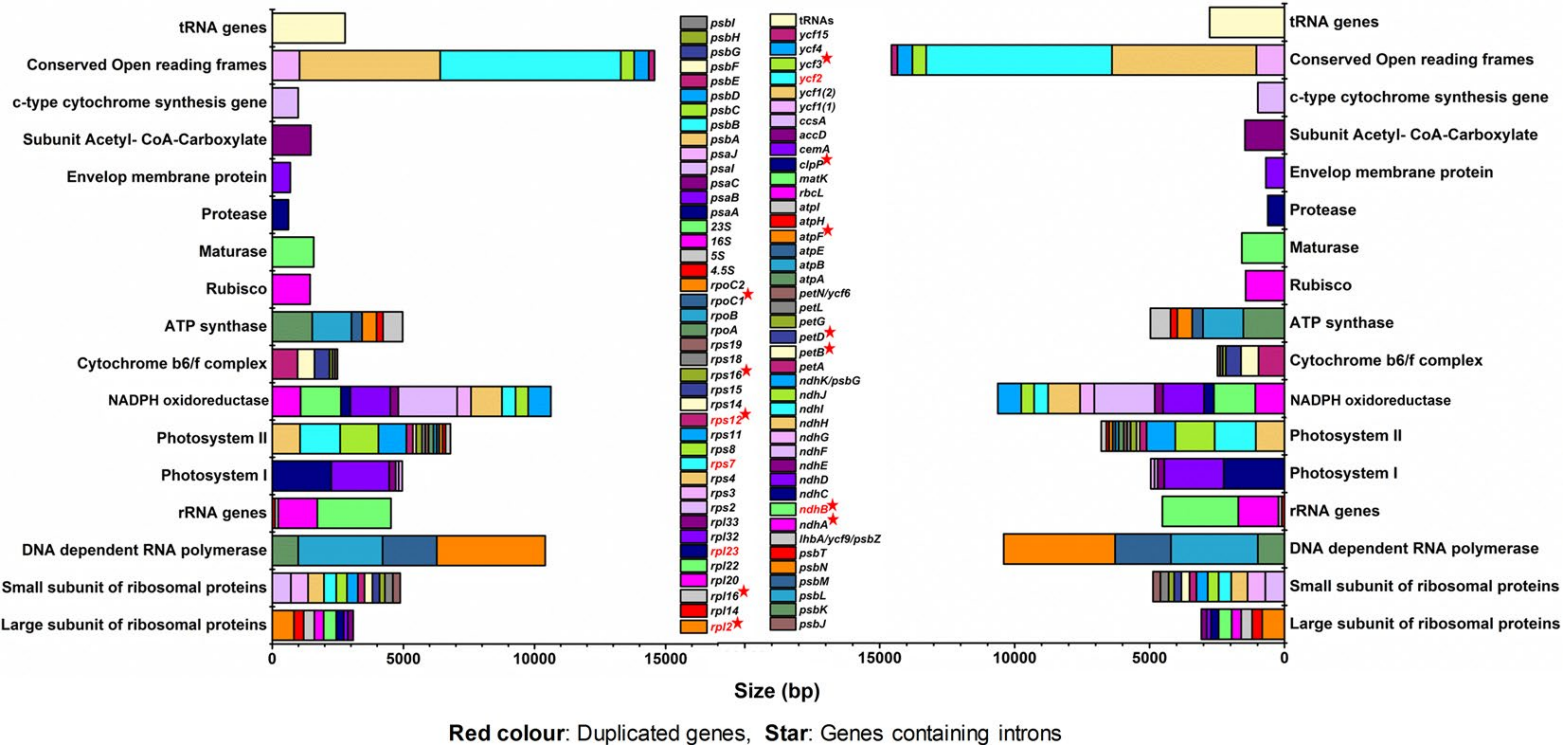
Gene contents of the *A*. *halleri* ssp. *gemmifera* and *A*. *lyrata* ssp. *petraea* chloroplast genomes, grouped by gene family. The colour of each gene is unique within its gene family. Horizontal axis indicates each box is proportional to the size of the gene (bp), including introns.

Both cp genomes were composed of 130 genes, including 86 protein-coding genes, eight ribosomal RNA (rRNA) genes and 37 transfer RNA (tRNA) genes (Figs 1, 2; Table 2). Five protein-coding genes (*rpl2*, *rpl23*, *ycf2*, *ndhB* and *rps7*) are duplicated and the inverted repeats contain truncated copies of *rps19*, *ycf1*, *ndhF* and duplicated exons of *rps12* (exon 2 and exon 3). The LSC region was composed of 62 protein-coding and 22 tRNA genes, whereas the SSC region was composed of 12 protein-coding genes and 1 tRNA gene. The protein-coding genes present in the *A*. *halleri* ssp. *gemmifera* and *A*. *lyrata* ssp. *petraea* genomes included nine genes for large ribosomal proteins (*rpl2*, 14, 16, 20, 22, 23, 32, 33, 36), 12 genes for small ribosomal proteins (*rps2*, 3, 4, 7, 8, 11, 12, 14, 15, 16, 18, 19), five genes for photosystem I (*psaA*, *B*, *C*, *I*, *J*), 15 genes related to photosystem II (*psbA*, *B*, *C*, *D*, *E*, *F*, *H*, *I*, *J*, *K*, *L*, *M*, *N*, *T*, *Z*), and six genes (*atpA*, *B*, *E*, *F*, *H*, *I*) for ATP synthase and the electron transport chain (Fig. 2, Table 1).

**Table 2:** List of genes in the *A*. *halleri* ssp. *gemmifera* and *A*. *lyrata* ssp. *petraea* chloroplast genomes.

Category	Group of genes	Name of genes
Self-replication	Large subunit of ribosomal proteins	*rpl2* ^*,a^ *, 14, 16* ^*^ *, 20, 22, 23* ^a^ *, 32, 33, 36*
	Small subunit of ribosomal proteins	*rps2*, *3*, *4*, *7* ^a^, *8*, *11*, *12* ^*,a^, *14*, *15*, *16* ^*^, *18*, *19*
	DNA dependent RNA polymerase	*rpoA*, *B*, *C1* ^*^, *C2*
	rRNA genes	*rrn16* ^a^, *rrn23* ^a^, *rrn4*.*5* ^a^, *rrn5* ^a^
	tRNA genes	*trnA-UGC* ^*,a^, *trnC-GCA*, *trnD-GUC*, *trnE-UUC trnF-GAA*, *trnfM-CAU*, trnG-UCC*, *trnH-GUG*, *trnI-CAU*, *trnI-GAU* ^*,a^, trnK-UUU*, *trnL-CAA*, trnL-UAA*, *trnL-UAG*, *trnM-CAU*, *trnN-GUU*, *trnP-GGG*, *trnP-UGG*, *trnQ-UUG*, *trnR-ACG*, *trnR-UCU*, *trnS-GCU*, *trnS-GGA*, *trnS-UGA*, *trnT-GGU*, *trnT-UGU*, *trnV-GAC*, trnV-UAC*, *trnW-CCA*, *trnY-GUA*
Photosynthesis	Photosystem I	*psaA*, *B*, *C*, *I*, *J*
	Photosystem II	*psbA*, *B*, *C*, *D*, *E*, *F*,*H*, *I*, *J*, *K*, *L*, *M*, *N*, *T*, *lhbA*
	NadH oxidoreductase	*ndhA* ^*^, *B* ^*,a^, *C*, *D*, *E*, *F*, *G*, *H*, *I*, *J*, *K*
	Cytochrome b6/f complex	*petA*, *B* ^*^, *D* ^*^, *G*, *L*, *N*
	ATP synthase	*atpA*, *B*, *E*, *F* ^*^, *H*, *I*
	Rubisco	*rbcL*
Other genes	Maturase	*matK*
	Protease	*clpP* ^*^
	Envelop membrane protein	*cemA*
	Subunit Acetyl- CoA-Carboxylate	*accD*
	c-type cytochrome synthesis gene	*ccsA*
	Conserved Open reading frames	*ycf1*, *2* ^a^, *3* ^*^, *4*

*Genes containing introns; ^a^Duplicated gene (Genes present in the IR regions).

Proteins are encoded by 51.80% (*A*. *halleri*) and 51.79% (*A*. *lyrata*) of the cp genomes, while rRNAs and tRNAs comprise 5.85% and 1.79%, respectively, of both genomes, with the remaining 40.5% made up of non-coding regions (Table 3). The total protein-coding sequences (CDS) were 80,019 and 80,013 bp in length in these two cp genomes, respectively, and composed of 86 protein-coding genes that code for 26,907 and 26,905 codons, respectively (Tables 3 and 4). The codon usage frequency was calculated for tRNA and protein-coding gene sequences in the *A*. *halleri* ssp. *gemmifera* (Table S3) and *A*. *lyrata* ssp. *petraea* (Table S4) cp genomes. In these cp genomes, leucine (10.7%) was the most common amino acid, while cysteine (1.2%) was least common. Furthermore, isoleucine, serine, glycine, arginine and alanine were present at 7.9, 7.5, 7.4, 6.5, and 6.1%, respectively.

**Table 3:** Comparison of coding and non-coding region size among twelve *Arabidopsis* species.

Region	*A. are*	*A. ceb*	*A.h. gem*	*A. l. pet*	*A. ped*	*A. tha*	*A. aren*	*A. cro*	*A. neg*	*A. pet*	*A. sue*	*A. ume*
**Protein Coding**												
Length (bp)	78561	78564	80019	80013	78540	79368	78648	78672	78675	78675	78666	78699
GC (%)	37.1	37.1	37	37	37.1	37	37.1	37	37.1	37.1	37	37.1
Length (%)	50.7	50.8	51.80	51.79	50.70	51.3	50.8	50.8	50.8	50.8	50.9	50.8
**tRNA**												
Length (bp)	2790	2790	2775	2775	2796	3325	2790	2790	2790	2791	2789	2791
GC (%)	52.6	52.6	52.2	52.3	52.5	49.2	52.6	52.6	52.6	52.6	52.5	52.6
Length (%)	1.80	1.80	1.79	1.79	1.80	2.15	1.80	1.80	1.80	1.80	1.80	1.80
**rRNA**												
Length (bp)	9050	9050	9050	9050	9050	8929	9050	9050	9050	9050	9050	9050
GC (%)	55.4	55.4	55.4	55.4	55.4	55.4	55.4	55.4	55.4	55.4	55.4	55.4
Length (%)	5.84	5.85	5.85	5.85	5.84	5.78	5.8	5.84	5.84	5.84	5.84	5.84
Intergenic	64470	64100	62629	62629	64509	62256	64302	64216	64284	64347	63861	64205
GC (%)	31.6	31.6	31.8	31.7	31.7	31	31.3	31.1	31.5	31.3	31.2	31.6
Length (%)	41.6	41.48	40.56	40.53	41.64	40.3	41.5	41.5	41.52	41.55	41.36	41.51

**A. are** = *A*. *arenosa;* **A. ceb** = *A*. *cebennensis;* **A. h. gem** = *A*. *halleri ssp*. *gemmifera;* **A. l. pet** = *A*. *lyrata* ssp. *petraea;* **A. ped** = *A*. *pedemontana*; **A. tha** = *A*. *thanliana;* **A. aren** = *A*. *arenicola;* **A. cro** = *A*. *croatica;* **A. neg** = *A*. *neglecta;* **A. pet** = *A*. *petrogenea;* **A. sue** = *A*. *suecia;* **A. ume** = *A*. *umezawana*.

**Table 4:** Base compositions in the *A*. *halleri* ssp. *gemmifera* (Ahg) and *A*. *lyrata* ssp. *petraea* (Alp) cp genome.

	T/U		C		A		G		Length (bp)	
	*A*. *h*. *gem*	*A*. *l*. *pet*	*A*. *h*. *gem*	*A*. *l*. *pet*	*A*. *h*. *gem*	*A*. *l*. *pet*	*A*. *h*. *gem*	*A*. *l*. *pet*	*A*. *h*. *gem*	*A*. *l*. *pet*
Genome	32.3	32.3	18.5	18.5	31.4	31.4	17.9	17.9	154473	154489
LSC	33.8	33.8	17.5	17.5	32.1	32.1	16.6	16.6	84197	84158
SSC	35.2	35.2	15.2	15.2	35.4	35.4	14.2	14.2	17739	17814
IR	28.8	28.8	22	22.0	28.9	29.0	20.3	20.3	26270	26259
tRNA	23.2	23.2	26.3	26.3	24.5	24.5	25.9	25.9	2775	2775
rRNA	22.3	22.3	27.7	27.7	22.3	22.3	27.7	27.7	9050	9050
Protein Coding genes	31.5	31.9	17.3	17.3	31	31.1	19.7	19.7	80019	80013
1st position	24.24	24.26	16.8	18.6	30.34	30.3	26.75	28.4	26907	26905
2nd position	33.05	33.03	20.2	20.2	28.83	28.8	17.84	17.8	26907	26905
3rd position	38.6	36.6	13.3	14.03	32.13	32.12	15.54	15.5	26907	26905

**A. h. gem** = *A*. *halleri ssp*. *gemmifera;* **A. l. pet** = *A*. *lyrata* ssp. *petraea*.

Among these, the most common codon used was ATT (1,077), encoding isoleucine, and the least common codons were CTG (1) in *A*. *lyrata* ssp. *petraea* and ATT (1) in *A*. *halleri* ssp. *gemmifera*, which encodes methionine. The AT content was 54.58%, 61.88%, and 70.73% at the 1st, 2nd, and 3rd codon positions within the *A*. *halleri* ssp. *gemmifera* CDS region (Table 4). In *A*. *lyrata* ssp. *petraea*, the AT content was 54.56%, 61.03%, and 68.72% at the 1st, 2nd, and 3rd codon positions within the CDS region (Table 4).

### Repeat Analysis and comparison of its distribution in *Arabidopsis* subspecies

**Figure 3:**
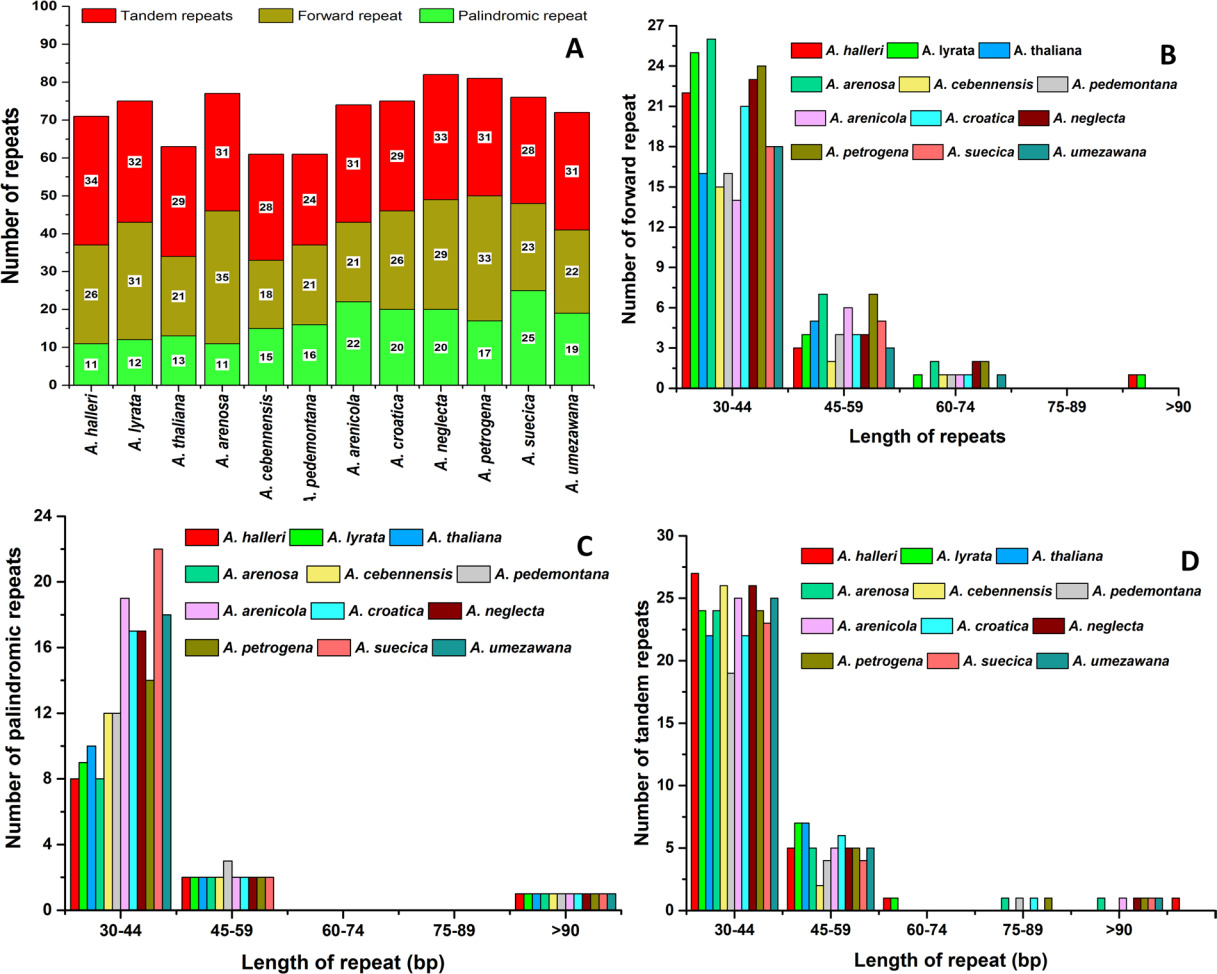
Analysis of repeated sequences in twelve *Arabidopsis* cp genomes. (**A**) Totals of three repeat types; (**B**) Frequency of forward repeats by length; (**C**) Frequency of palindromic repeats by length; (**D**) Frequency of tandem repeats by length.

A total of 71 and 75 repeats were detected in the *A*. *halleri* ssp. *gemmifera* and *A*. *lyrata* ssp. *petraea* genomes, respectively, using REPuter, including direct, reversed and palindromic repeats (Tables S5, S6). In these cp genomes, the repeat analysis detected 11 and 12 palindromic repeats, 26 and 31 forward repeats, and 34 and 32 tandem repeats in *A*. *halleri* ssp. *gemmifera* and *A*. *lyrata* ssp. *petraea*, respectively (Fig. 3). Among these, 22 forward repeats were 30–44 bp in length, 5 tandem repeats were of the same length, and 27 were 15–29 bp in length (Fig. 3A–D). Similarly, in *A*. *halleri* ssp. *gemmifera*, 8 palindromic repeats were 30–44 bp, and 2 repeats were 45–59 bp in length (Fig. 3D). However, in *A*. *lyrata* ssp. *petraea*, 25 forward repeats and nine palindromic repeats were 30–44 bp in length, and 27 tandem repeats were 15–29 bp in length (Fig. 3B–D).

Similarly, 63, 77, 61, 61, 74, 75, 82, 81, 76 and 72 repeat pairs were found in previously reported *A*. *thaliana*, *A*. *arenosa*, *A*. *cebennensis*, *A*. *pedemontana*, *A*. *arenicola*, *A*. *croatica*, *A*. *neglecta*, *A*. *petrogena*, *A*. *suecica* and *A*. *umezawana* genomes, respectively (Fig. 3A). This suggests that *A*. *halleri* and *A*. *lyrata* are more similar to *A*. *arenosa*, *A*. *croatica*, *A*. *suecica* and *A*. *umezawana* with respect to repeats. The lengths of direct and palindromic repeats in the *A*. *halleri* ssp. *gemmifera* and *A*. *lyrata* ssp. *petraea* cp genomes were much shorter, ranging from 30 to 101 bp (Tables S5, S6). In *A*. *halleri* ssp. *gemmifera*, a minority of repeats were found in introns (7.89%), while the majority were located in IGS (55.2%) and coding sequence (CDS) regions (36.8%), while in *A*. *lyrata* ssp. *petraea* these values were 33.3%, 11.90% and 52.3%, respectively (Tables S5 and S6).

### Simple Sequence Repeat (SSR) Analysis and comparison

**Figure 4:**
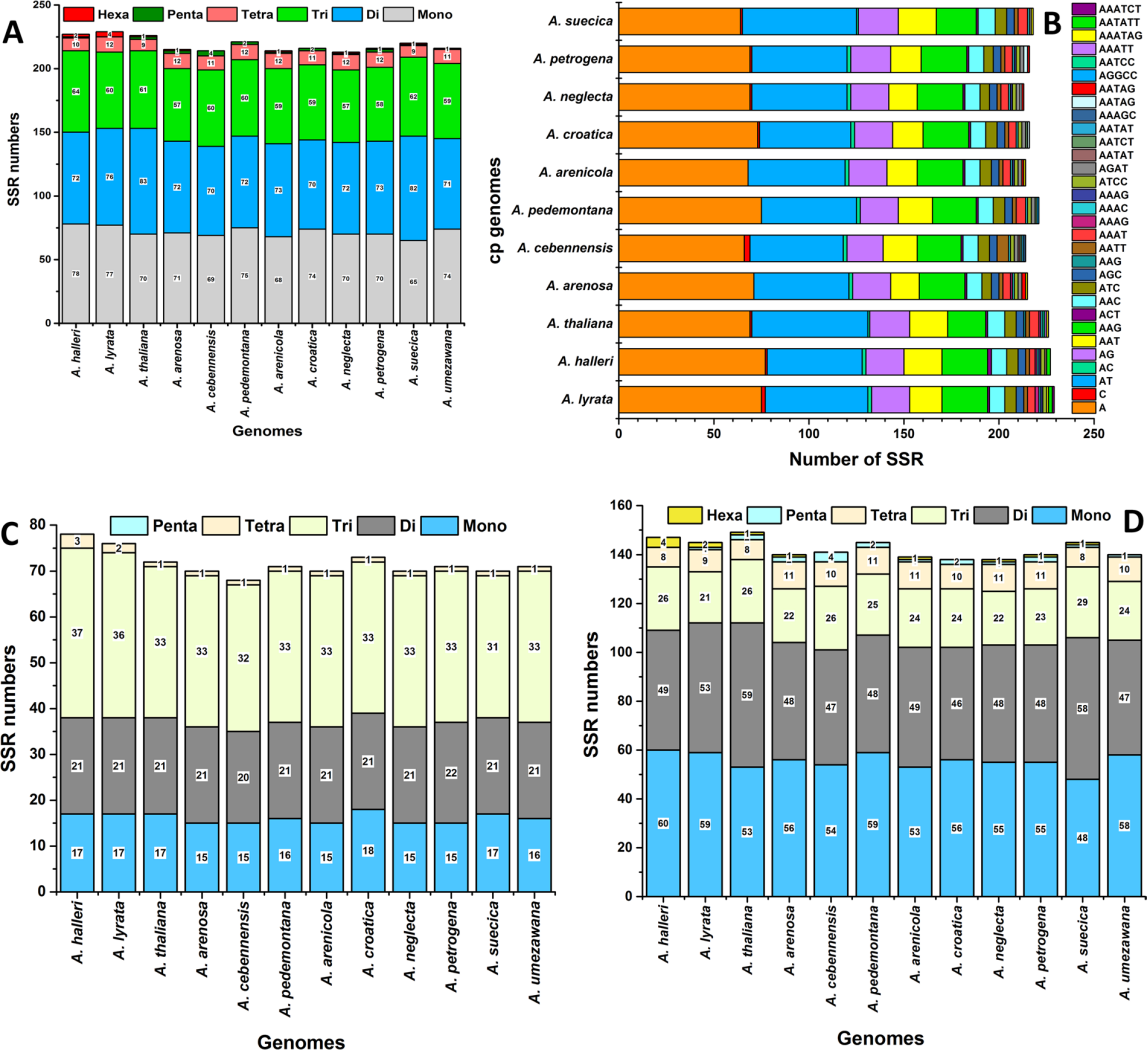
Analysis of simple sequence repeats (SSR) in the twelve *Arabidopsis* cp genomes. (**A**) Number of different SSR types detected in the six genomes; (**B**) Frequency of identified SSR motifs in different repeat class types; (**C**) Frequency of identified SSRs in coding regions; (**D**) Frequency of identified intergenic regions.

In this study, we detected perfect SSRs in the cp genomes of *A*. *halleri* ssp. *gemmifera* and *A*. *lyrata* ssp. *petraea* cp together with ten other *Arabidopsis* species (Fig. 4A–D). Certain parameters were set because SSRs of 10 bp or longer are prone to slipped strand mis-pairing, which is believed to be the main mutational mechanism of SSR polymorphisms. A total of 227 and 229 microsatellites were found in the *A*. *halleri* ssp. *gemmifera* and *A*. *lyrata* ssp. *petraea* cp genomes based on SSR analysis, respectively (Fig. 4A). Among these, 78 and 76 were found in coding regions, while 144 and 148 microsatellites were found in intergenic regions (Fig. 4A). Similarly, 226, 215, 214, 221, 214, 216, 213, 216, 220, and 216 SSRs were detected in *A*. *thaliana*, *A*. *arenosa*, *A*. *cebennensis*, *A*. *pedemontana*, *A*. *arenicola*, *A*. *croatica*, *A*. *neglecta*, *A*. *petrogena*, *A*. *suecica* and *A*. *umezawana*, respectively (Fig. 4A). The majority of the SSRs in these cp genomes consist of mono- and dinucleotide repeat motifs, varying from 65 in *A*. *suecica* to 78 in *A*. *halleri* ssp. *gemmifera* for mononucleotide repeats, while dinucleotide repeats varied from 70 in *A*. *cebennensis* and *A*. *croatica* to 83 in *A*. *thaliana* (Fig. 4A). Trinucleotide SSRs are the second most common, ranging from 57 in *A*. *arenosa* and *A*. *neglecta* to 64 in *A*. *halleri* ssp. *gemmifera*. Furthermore, two pentanucleotide SSRs are present in *A*. *thaliana*, *A*. *arenosa*, *A*. *pedemontana*, *A*. *croatica*, and *A*. *petrogena*, with one present in *A*. *halleri* ssp. *gemmifera*, *A*. *arenicola*, *A*. *neglecta*, and *A*. *umezawana*, and four in *A*. *cebennensis*. Additionally, four hexanucleotide repeats were found in *A*. *lyrata* ssp. *petraea*, one in *A*. *thaliana*, *A*. *arenosa*, *A*. *arenicola*, *A*. *neglecta*, *A*. *suecica*, and two in *A*. *halleri* ssp. *gemmifera* using our search criterion (Fig. 4A, Table S7). In *A*. *halleri* ssp. *gemmifera* and *A*. *lyrata* ssp. *petraea*, most mononucleotide SSRs were A (98.7%, 97.4%) motifs, with the majority of dinucleotide SSRs being A/T (71.05%, 69.44%) and A/G (27.77%, 26.31%) motifs (Fig. 4B, Tables S7 and S8).

### Structural comparative assessment of cp genomes in *Arabidopsis*

**Figure 5:**
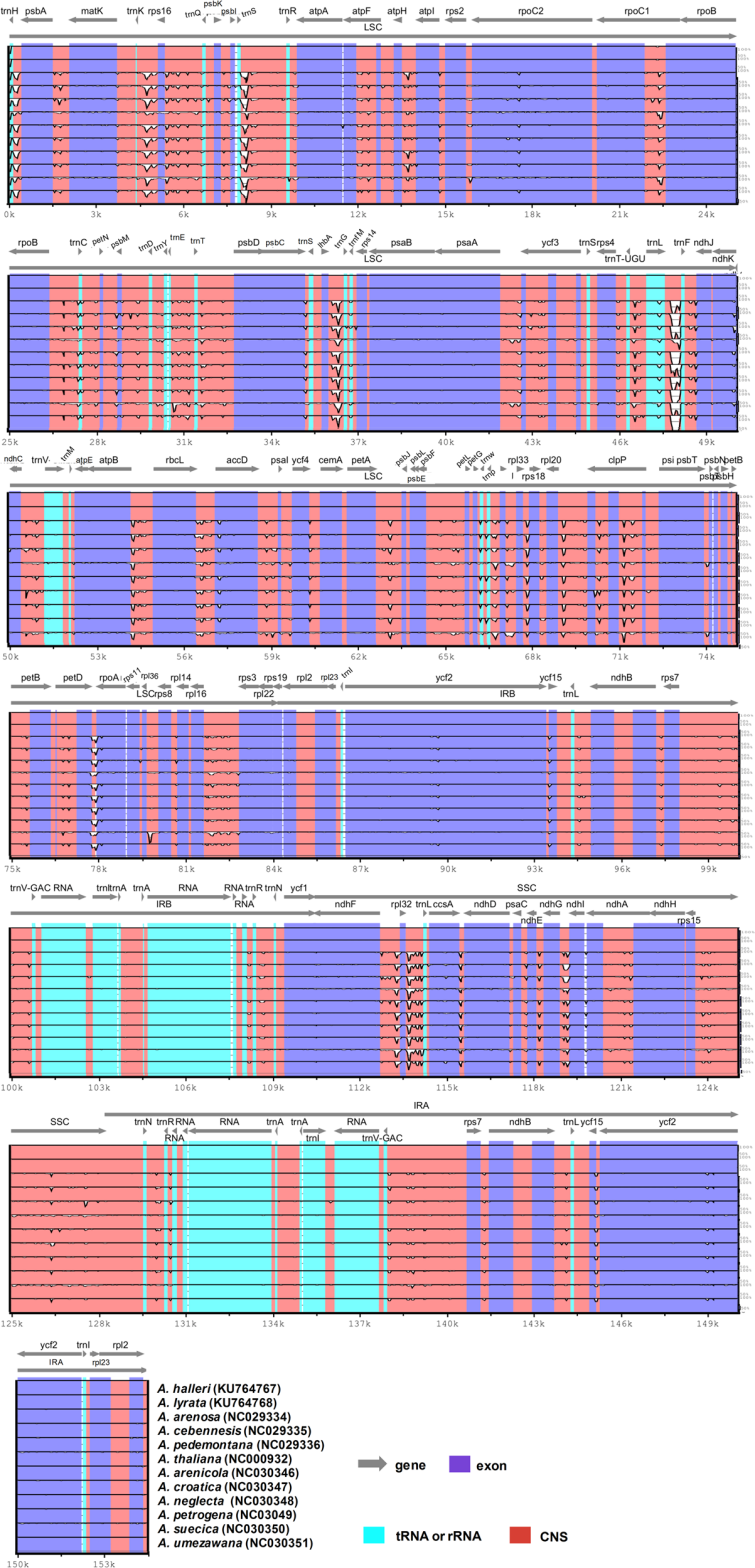
Alignment visualization of the twelve *Arabidopsis* chloroplast genome sequences. VISTA-based identity plot showing sequence identity among the six-species using *A*. *halleri* ssp. *gemmifera* as a reference. Vertical scale indicates the percentage of identity, ranging from 50% to 100%. Horizontal axis indicates the coordinates within the chloroplast genome. Arrows indicate the annotated genes and their transcriptional direction. The thick black lines show the inverted repeats (IRs) in the chloroplast genomes.

Ten complete cp genomes within the genus *Arabidopsis* (*A*. *thaliana*, *A*. *arenosa*, *A*. *cebennensis*, *A*. *pedemontana*, *A*. *arenicola*, *A*. *croatica*, *A*. *neglecta*, *A*. *petrogena*, *A*. *suecica* and *A*. *umezawana*) were selected for comparison with *A*. *halleri* ssp. *gemmifera* (154,473 bp) and *A*. *lyrata* ssp. *petraea* (154,489 bp). The genome size of *A*. *pedemontana* (154,895 bp) is the largest of these, and this difference was mostly attributed to variation in the length of the LSC region (Table 1), as reported previously in angiosperms cp genomes. Analysis of genes with known functions showed that *A*. *halleri* ssp. *gemmifera* and *A*. *lyrata* ssp. *petraea* shared 70 protein-coding genes with the other ten *Arabidopsis* species cp genomes. The number of unique genes found in these cp genomes was 79 (Fig. 5).

Pairwise cp genomic alignment of *A*. *halleri* ssp. *gemmifera* and *A*. *lyrata* ssp. *petraea* with ten other *Arabidopsis* cp genomes uncovered a high degree of synteny. Annotations from cp genomes of *A*. *halleri* ssp. *gemmifera* and *A*. *lyrata* ssp. *petraea* were used as a reference to plot. The sequences of eleven *Arabidopsis* species cp genomes were compared using mVISTA (Fig. 5 and Fig. S1). Furthermore, we compared the *Arabidopsis* cp genomes and calculated the average pairwise sequence divergence among these twelve species (Table S9). Of these genomes, *A*. *halleri* ssp. *gemmifera* and *A*. *lyrata* ssp. *petraea* exhibited 0.0039 and 0.0045 average sequence divergence, respectively, and both species were highly divergent (0.0123 and 0.0128, respectively) with *A*. *thaliana* (Table S9). For both *A*. *halleri* ssp. *gemmifera* and *A*. *lyrata* ssp. *petraea*, the lowest average sequence divergences were found with *A*. *umezawana* (0.0005) and *A*. *arenicola* (0.0019). Additionally, the ten most divergent genes among these *Arabidopsis* cp genomes were *rps8*, *psbK*, *petD*, *psbM*, *ndhD*, *E*, *accD*, *rps12*, *rpl33* and *atpH* (Figs S2 and S3). The highest average sequence distance of *A*. *halleri* ssp. *gemmifera* from the other cp genomes was observed in *psbM* (0.0292), followed by *rpl22* (0.0210), with *A*. *thaliana* and *A*. *suecica*, respectively (Fig. S2). In *A*. *lyrata* ssp. *petraea* the highest average sequence distances were observed for *psbK* (0.021), rpl22 (0.021) and *psbM* (0.0194) (Fig. S3).

### Inverted repeat (IR) contraction and expansion across two subspecies

**Figure 6:**
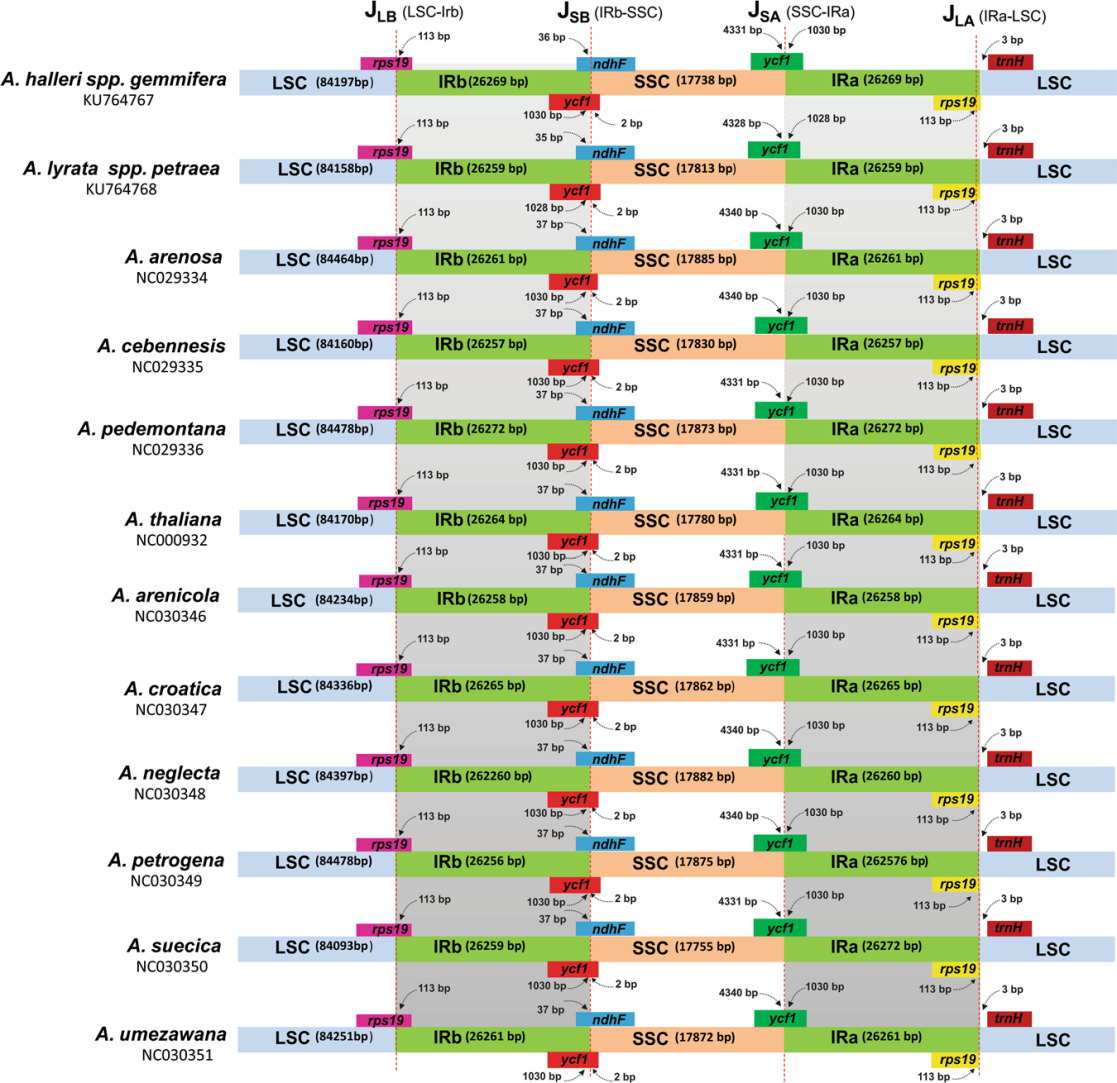
Comparison of border distance between adjacent genes and junctions of LSC, SSC, and two IR regions among the chloroplast genomes of twelve *Arabidopsis* species. Boxes above or below the main line indicate the adjacent border genes. The figure is not to scale with respect to sequence length and only shows relative changes at or near the IR/SC borders.

A detailed comparison was performed of four junctions (J_LA_, J_LB_, J_SA_ and J_SB_) between the two IRs (IRa and IRb) and the two single-copy regions (LSC and SSC) among *A*. *thaliana*, *A*. *arenosa*, *A*. *cebennensis*, *A*. *pedemontana*, *A*. *arenicola*, *A*. *croatica*, *A*. *neglecta*, *A*. *petrogena*, *A*. *suecica* and *A*. *umezawana* in comparison to *A*. *halleri* ssp. *gemmifera* and *A*. *lyrata* ssp. *petraea* (Fig. 6). We carefully analysed and compared the exact IR border positions and the adjacent genes among the *Arabidopsis* species cp genomes (Fig. 6). In this study, despite the similar length of the IR regions in *A*. *halleri* ssp. *gemmifera* and *A*. *lyrata* ssp. *petraea* with those in the other ten *Arabidopsis* species (from 26,256 bp in *A*. *petrogena* to 26,272 bp in *A*. *pedemontana*), some IR contraction and expansion was observed. The LSC/IRb junction was located in the *rps19* region in all *Arabidopsis* species plastid genomes, which extended 113 bp into the IRb region in all genomes. The IRa ends up with the truncated copy of 113 bp of *rps19* gene in all twelve species. Similarly, the *trnH* gene was located in LSC region, 3 bp away from the IRa/LSC border across compared genomes. The *ndhF* gene was located in the J_SB_ border and extended 35 and 36 bp in *A*. *lyrata* ssp. *petraea* and *A*. *halleri* ssp. *gemmifera*, respectively, and 37 bp in the other *Arabidopsis* species in the IRb region. The pseudogene *ycf* extended 3 bp into the SSC region at the border of J_SB_ for *A*. *halleri* ssp. *gemmifera*, and 2 bp in *A*. *thaliana*, *A*. *arenosa*, *A*. *cebennensis*, *A*. *pedemontana*, *A*. *arenicola*, *A*. *croatica*, *A*. *neglecta*, *A*. *petrogena*, *A*. *suecica* and *A*. *umezawana*. A similar gene composition was found in the J_SA_ border. In *A*. *lyrata* ssp. *petraea*, the *ycf1* gene was located 1030 and 1028 bp in the IR regions, and in other *Arabidopsis* species, this distance was 1030 bp away from the J_SA_ border in IR.

### Phylogenetic analysis of *A*. *halleri* ssp. *gemmifera* and *A*. *lyrata* ssp. *petraea*

**Figure 7:**
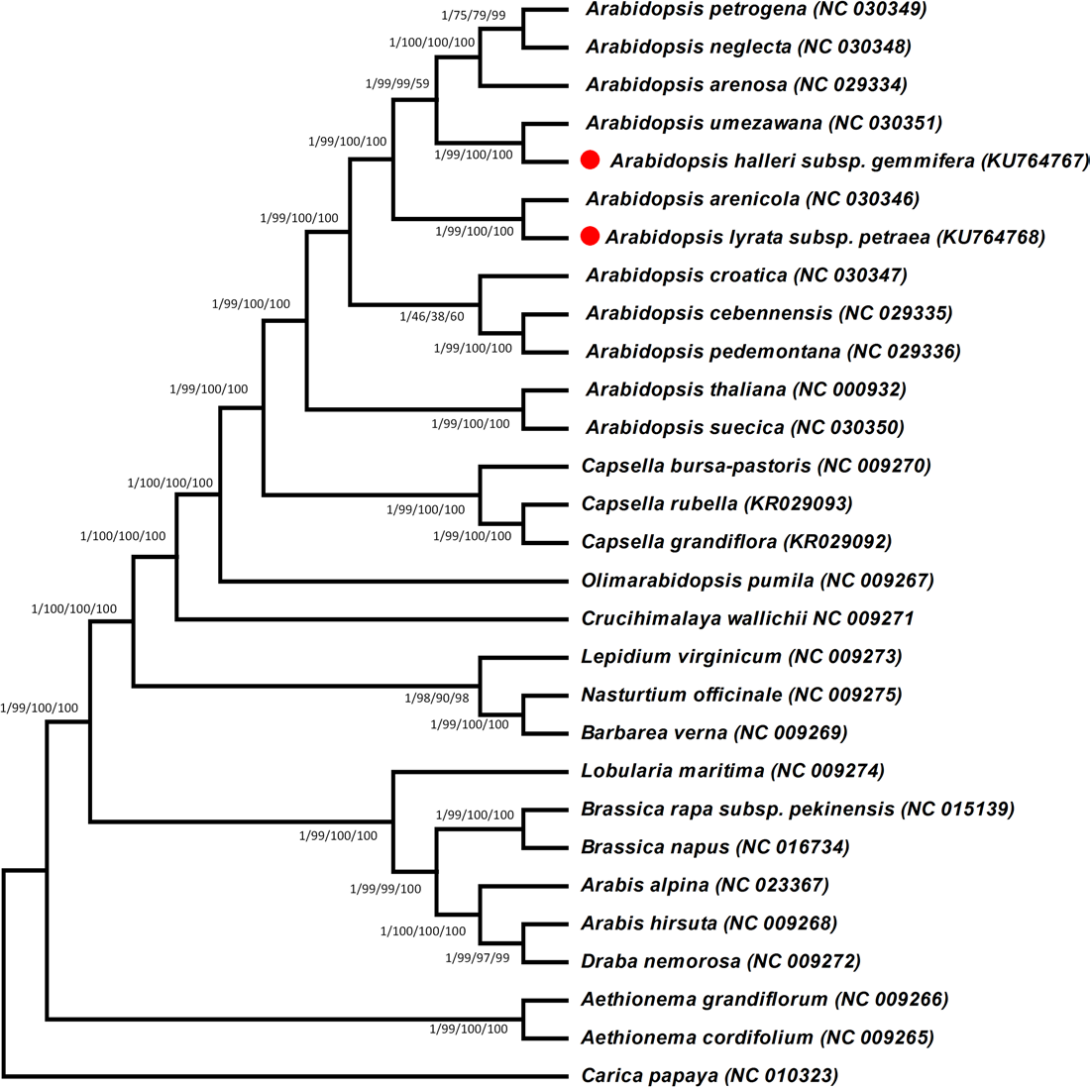
Phylogenetic trees were constructed for twenty-eight species from the family Brassicaceae using several different methods, and the tree shown is for the 70 shared protein coding genes. The following four different methods were used for the 70 shared genes data set: Bayesian inference (BI), maximum parsimony (MP), maximum likelihood (ML) and neighbour-joining (NJ). Numbers above the branches are the posterior probabilities of BI and bootstrap values for NJ, MP and ML. Stars represent the positions of *A*. *halleri* ssp. *gemmifera* and *A*. *lyrata* ssp. *petraea*.

In this study, the phylogenetic position of *A*. *halleri* ssp. *gemmifera* and *A*. *lyrata* ssp. *petraea* within the family *Brassicaceae* was established by analysing multiple alignments of complete cp genomes and 70 shared genes of 29 Brassicaceae members representing 12 genera (Fig. 7 and Fig. S4). *Carica papaya* was set as the outgroup. Phylogenetic analyses using Bayesian inference (BI), maximum parsimony (MP), maximum likelihood (ML) and neighbour-joining (NJ) were performed. The results revealed that complete cp genomes and 70 shared genes of *A*. *halleri* ssp. *gemmifera* and *A*. *lyrata* ssp. *petraea* contain the same phylogenetic signals; the complete genome sequence and the 70 shared genes (from all species) generated phylogenetic trees with identical topologies (Fig. 7 and Fig. S4). Maximum likelihood (ML) analysis revealed 22 out of 26 nodes with bootstrap values ≥ 99%, and most of these nodes had 100% bootstrap values. In these phylogenetic trees based on the entire genome data set and the 70 shared genes, *A*. *halleri* ssp. *gemmifera* formed a single clade with *A*. *umezawana*, and *A*. *lyrata* ssp. *petraea* formed a single clade with *A*. *arenicola* for high Bayesian inference (BI), and bootstrap support using four different methods (Fig. 7 and Fig. S4).

## Discussion

This study reports the complete chloroplast genomes of *A*. *halleri* ssp. *gemmifera* and *A*. *lyrata* ssp. *petraea*, ranging from 154.4~154.5 kbp in length. Both cp genomes exhibit a typical quadripartite structure, as reported for other angiosperms. Both genomes encode ~130 genes, including 85 protein-coding genes, 8 ribosomal RNA genes and 37 transfer RNA genes, with 227 to 229 microsatellites distributed randomly throughout their genomes, respectively. In addition, approximately 26/31 forward, 34/32 tandem and 11/12 palindromic repeats were found in both cp genomes. This conformed with the protein coding genes found in other *Brassicaceae* members, such as *A*. *thaliana* ^16^, *Brassica nigra*, *Brassica oleracea*,^41^ and *Capsella rubella* ^42^. Among the coding genes, *rps12* is an unequally divided gene, with its 5′ terminal exon located in the LSC region, while two copies of the 3′ terminal exon and intron are located in IRs, which is similar to other angiosperm cp genomes. The pseudogene *ycf1* was located in the boundary regions between IRb and SSC, leading to incomplete duplication of the gene within IR regions. We found twelve intron-containing genes in both of the cp genomes, nine of which contained one intron, whereas the genes *ycf3*, *clpP*, and *rps12* contained two introns. The gene *ndhA* had the longest intron in both *A*. *halleri* ssp. *gemmifera* (1,089 bp) and *A*. *lyrata* ssp. *petraea* (1,084) (Tables S10 and 11). This intron plays an important role in the regulation of gene expression, and some recent research revealed that various introns improve exogenous gene expression at specific positions. Therefore, the intron can be a valuable tool to improve transformational efficiency^43^. It was observed that *ycf1*, *ycf2* ^44, 45^, *rpl23* ^46^ and *accD* ^47, 48^ are often absent in plants^46^, but they were detected in the reported *Arabidopsis* cp genomes. The pair of genes *atpB-atpE* was observed to overlap each other by ~4 bp. However, *psbC-psbD* had a 92 bp overlap in the *A*. *halleri* ssp. *gemmifera* and *A*. *lyrata* ssp. *petraea* cp genomes, while this overlap was 53 bp in *A*. *thaliana*, 17 bp in *A*. *arenosa*, 53 bp in *Gossypium* ^49^ and 52 bp in *Camellia* cp genomes^50^. Similar ratios of amino acids were observed in comparison to the previously reported cp genomes^50–52^. The preference for a high AT content at the 3^rd^ codon position is consistent with the A and T concentrations reported in various terrestrial plant cp genomes^17, 51, 53, 54^.

We found 71 and 75 repeats in the cp genomes of *A*. *halleri* ssp. *gemmifera* and *A*. *lyrata* ssp. *petraea*, respectively, which included reversed, direct and palindromic repeats. Repeat sequences are very helpful in phylogenetic studies and play a role in genome rearrangements^53, 55^. Furthermore, analyses of the various cp genomes concluded that repeat sequences are essential to inducing indels and substitutions^56^. The length of direct and palindromic repeats in the *A*. *halleri* ssp. *gemmifera* and *A*. *lyrata* ssp. *petraea* cp genomes were much shorter, ranging from 30–101 bp., and similar results have been previously reported in *Camellia*, which has an 82 bp repeat. However, much longer repeats have been observed, such as the 132 bp and 287 bp repeats found in *Poaceae* and *Fabaceae*, respectively^57^. Previous reports suggested that sequence variation and genome rearrangement occur due to slipped strand mispairing and the improper recombination of these repeat sequences^58, 59^. Furthermore, the presence of these repeats indicates that the region is a crucial hotspot for genome reconfiguration^59^. Additionally, these repeats are an informative source for developing genetic markers for *A*. *halleri* ssp. *gemmifera* and *A*. *lyrata* ssp. *petraea* for phylogenetic and population studies^55^.

During genome annotation, we noted perfect SSRs in *A*. *halleri* ssp. *gemmifera* and *A*. *lyrata* ssp. *petraea* compared to the ten other *Arabidopsis* species cp genomes. SSRs usually have a higher mutation rate compared to other neutral DNA regions due to slipped DNA strands. These are present in the cp genome at the highest diversity in copy numbers and are important molecular markers for plant population genetics, evolutionary, and ecological studies^60^. We looked for SSRs of 10 bp or longer, as these have been suggested to be prone to slipped strand mispairing. This is believed to be the main mutational mechanism for SSR polymorphisms^61, 62^. From our SSR analysis, 227 and 229 microsatellites were found in the *A*. *halleri* ssp. *gemmifera* and *A*. *lyrata* ssp. *petraea* cp genomes, respectively. In addition, 226, 215, 214, 221, 214, 216, 213, 216, 220, and 216 SSRs were detected in *A*. *thaliana*, *A*. *arenosa*, *A*. *cebennensis*, *A*. *pedemontana*, *A*. *arenicola*, *A*. *croatica*, *A*. *neglecta*, *A*. *petrogena*, *A*. *suecica* and *A*. *umezawana*, respectively.

These findings are consistent with the previous observation that the SSRs of cp genomes are dominated by ‘A’ or ‘T’ mononucleotide repeats^16, 63^. Mononucleotide, pentanucleotide and hexanucleotides repeats were also composed of ‘A’ or ‘T’ at greater frequencies, which reflects a biased base composition, with an overall A-T richness in the cp genomes^64, 65^. Our findings are comparable to previous reports that showed that SSRs found in the cp genome are generally composed of polythymine (polyT) or polyadenine (polyA) repeats and infrequently contain tandem cytosine (C) and guanine (G) repeats^65^. Therefore, these SSRs contribute to the ‘AT’ richness of the *A*. *halleri* ssp. *gemmifera* and *A*. *lyrata* ssp. *petraea* cp genomes, as previously reported for different species^52, 66^. The current analysis showed that approximately 69% (*A*. *halleri* ssp. g*emmifera*) and 77% (*A*. *lyrata* ssp. *petraea*) of SSRs were detected in non-coding regions. These results agree with previous reports that SSRs are unevenly distributed in cp genomes and might provide more information for selecting effective molecular markers for the detection of intra- and interspecific polymorphisms^67, 68^.

Our results reveal that both the *A*. *halleri* ssp. *gemmifera* and *A*. *lyrata* ssp. *petraea* cp genomes share high sequence similarity with all ten *Arabidopsis* species. However, relatively lower identity was also observed with these species in several comparable genomic regions. In addition, similar to previously reported cp genomes^51, 52, 69–71^, the LSC and SSC regions were less similar than the two IR regions in all *Arabidopsis* species cp genomes. Similar results were reported previously in various higher plant cp genomes, which suggests that the lower sequence divergence in the IR regions compared to the SC and LSC regions is possibly due to copy correction between IR sequences by gene conversion^72^. Furthermore, the non-coding regions showed greater divergence than the coding regions. The divergent regions included *trnK*-*rps16*, *rpoC1*, *TrnL*-*TrnF*, *atpB*-*rbcL*, *accD*, *petA*-*psbJ*, *petD*-*rpoA*, *ccsA*, *rpl33*, *rps12*, *psbM*, *ndhD* and *ycf2*. Similar results for these genes were reported previously^51, 52^, and these results also confirm similar differences among various coding regions in the analysed species suggested by Yang *et al*.^73^. These results are consistent with previous reports that these divergent genes are mostly present in LSC regions and show a trend towards more rapid evolution^52^.

Regarding the IR regions, the expansion and contraction at the borders are the main reasons for size variations among cp genomes, and it plays a vital role in evolution^71, 74, 75^. A detailed comparison between the two IRs and two single-copy regions was performed among *A*. *thaliana*, *A*. *arenosa*, *A*. *cebennensis*, *A*. *pedemontana*, *A*. *arenicola*, *A*. *croatica*, *A*. *neglecta*, *A*. *petrogena*, *A*. *suecica* and *A*. *umezawana* in comparison to *A*. *halleri* ssp. *gemmifera* and *A*. *lyrata* ssp. *petraea*. In *A*. *halleri* ssp. *gemmifera* and *A*. *lyrata* ssp. *petraea*, the *ycf1* gene is located 1030 and 1028 bp in the IR regions, respectively. This gene is the second largest gene in the plastid genome and encodes a protein of approximately 1,800 amino acids. Recently, researchers reported that *ycf1* is essential for plant viability and encodes Tic214, a vital component of the *Arabidopsis* TIC complex^76, 77^.

Recently, Dong *et al*. found that two regions of the plastid gene *ycf1* were highly variable in flowering plants^77^. Specifically, the *ycf1* (pseudogene) located in the IRb region is conserved, while the *ycf1* in the SSC is highly variable. This region of the ycf1 gene is more variable than *matK* in most taxa investigated thus far^44, 45^. Therefore, researchers used the two regions of *ycf1* as a new tool to solve phylogenetic problems at the species level and for DNA barcoding of some closely related flowering plant species^78–81^. Furthermore, two regions within *ycf1*, *ycf1a and ycf1b* have been predicted to have the highest nucleotide diversity (π) at the species level within angiosperm plastid genomes^77, 82^.

Chloroplast genomes have shown substantial power in studies of phylogenetics, evolution and molecular systematics. During the last decade, there have been many analyses to address phylogenetic questions at deep nodes based on comparisons of multiple protein-coding genes^83, 84^ and complete cp genome sequences that enhance our understanding of enigmatic evolutionary relationships among angiosperms^38^. The genus *Arabidopsis* has been estimated to comprise at least 9 species and 6 subspecies^85^ or up to 13 species and 9 subspecies^86^ depending on the taxonomic approach and the identifier. According to the Hohmann *et al*.^38^ taxonomy, the genus *Arabidopsis* has been proposed to consist of as many as 26 taxa, including the model plant *A*. *thaliana* ^34, 86^. Continued efforts have enhanced our ability to differentiate lineages and to understand the genomic structure and phylogenetic relationships of *Arabidopsis* species^34^. Previous evolutionary relationships among different *Arabidopsis* genomes and species were estimated using nuclear and chloroplast DNA^87, 88^ restriction fragment-length polymorphisms, but complete genome sequencing provides more detailed insights^34^. Recently, Novikova *et al*.^34^ reported Illumina sequencing and phylogenetic analysis based on polymorphism data and chloroplast genome data for 26 taxa, including those presented in the current study, that constitute the genus *Arabidopsis* ^34^. In our study, the phylogenetic positions of *A*. *halleri* ssp. *gemmifera* and *A*. *lyrata* ssp. *petraea* within the family *Brassicaceae* were established by sequencing complete cp genomes and analysing 70 shared genes of 29 *Brassicaceae* members, representing 12 genera. The phylogenetic analysis showed that the complete cp genomes and the 70 shared gene data set exhibit identical phylogenetic signals. In both the entire genome data set and the 70 shared genes data set, *A*. *halleri* ssp. *gemmifera* forms a single clade with *A*. *umezawana*, and *A*. *lyrata* ssp. *petraea* forms a single clade with *A*. *arenicola*. Similar results were described by Hohmann *et al*.^38^ based on *trnLF* and ribosomal ITS data, finding that *A*. *halleri* ssp. *gemmifera* is a sister to *A*. *umezawana*. The results also confirmed a previous report by Hohmann *et al*.^38^ reporting that *A*. *lyrata* ssp. *petraea* and *A*. *arenicola* are close relatives and that *A*. *arenicola* probably originated postglacially from an *A*. *lyrata* population^37^. Furthermore, these results are also in broad agreement with previous results reported by Novikoa *et al*.^34^, where *A*. *halleri* ssp. *gemmifera* was most closely related to *A*. *umezawana* and *A*. *lyrata* ssp. *petraea* formed a clade with *A*. *arenicola* ^34^.

In conclusion, we assembled and analysed the complete chloroplast genomes of *A*. *halleri* ssp. *gemmifera* and *A*. *lyrata* ssp. *petraea* and compared them with other *Arabidopsis* species for the first time. The genome organization, gene order, GC content and codon usage were similar to those of previously reported cp genomes from the genus *Arabidopsis*. The location and distribution of repeat sequences was determined, and sequence divergences of cp genomes and 70 shared genes were calculated with related species. The phylogenetic analysis based on whole cp genomes and 70 shared genes yielded identical phylogenetic trees, with *A*. *halleri* ssp. *gemmifera* and *A*. *lyrata* ssp. *petraea* forming single clades with *A*. *umezawana* and *A*. *arenicola*, respectively.

## Materials and Methods

### Genome Sequencing and Assembly

A standard protocol of DNA extraction was followed, as described in detail by Hu *et al*.^24^. Pure DNA was sequenced on an Illumina HiSeq. 2000. A total of 63,528,604 and 67,938,537 raw reads were generated for *A*. *halleri* ssp. *gemmifera* and *A*. *lyrata* ssp. *petraea*, respectively, which were then trimmed and filtered using CLC Genomics Workbench v7.0 (CLC Bio, Aarhus, Denmark). Then, CLC Genomics Workbench v7.0 (CLC Bio, Aarhus, Denmark) was used for *de novo* genome assembly. Different k-mer sizes were evaluated, and a k-mer size of 66 provided the best results in terms of minimum members and longest average length of scaffolds. These parameters were used to generate the final assembly. Then, the resulting contigs were compared against the *A*. *thaliana* chloroplast genome using BLASTN with an E-value cutoff of 1e-5. Six and seven contigs of *A*. *halleri* ssp. *gemmifera* and *A*. *lyrata* ssp. *petraea*, respectively, were identified and were temporarily arranged based on their mapping positions on the reference genome. Then, primers were designed (Table S1) based on the sequence at the ends of the adjacent contigs. PCR amplification and subsequent DNA sequencing were used to fill the gaps. PCR amplification was performed in a total volume of 20 μl containing 1 × reaction buffer, 0.1 μl Taq DNA Polymerase, 0.4 μl dNTP (10 mM) and 1 μl (10 ng/μl) of DNA. The PCR programme was composed of an initial denaturation at 95 °C for 5 min followed by 32 cycles at 95 °C for 30 s, 60 °C for 20 s and 72 °C for 30 s, with a final extension step at 72 °C for 5 min. After incorporation of the Sanger sequencing results, the completed cp genome was used as a reference to map the initial short reads to refine the assembly based on maximum sequence coverage.

### Genome Annotation and Sequence Architecture

A program (DOGMA) was used to annotate the *A*. *halleri* ssp. *gemmifera* and *A*. *lyrata* ssp. *petraea* cp genomes^89^. The annotation results were checked manually, and codon positions were adjusted by comparing to homologues from the database *A*. *thaliana* cp genome. All transfer RNAs were verified using tRNAscan-SE version 1.21^90^ using the default settings. OGDRAW^91^ was used to illustrate structural features of the *A*. *halleri* ssp. *gemmifera* and *A*. *lyrata* ssp. *petraea* cp genomes. The relative synonymous codon usage (RSCU) was determined using MEGA 6.0^92^ to examine deviations in synonymous codon usage by avoiding the influence of amino acid composition. The software mVISTA was used in Shuffle-LAGAN mode to compare the whole genome variations of *A*. *halleri* ssp. *gemmifera* and *A*. *lyrata* ssp. *petraea* genome with four other cp genomes using the *A*. *halleri* ssp. *gemmifera* and *A*. *lyrata* ssp. *petraea* annotation as a reference^93^.

### Characterization of Repeat sequence and SSRs

We used REPuter to identify repeat sequences, including palindromic, reverse, and direct repeats, within the cp genome^94^. The following settings for repeat identification were used in REPuter: 1) Hamming distance of 3, 2) 90% or greater sequence identity, and 3) a minimum repeat size of 30 bp. Phobos version 3.3.12^95^ was used to detect (SSRs) within the cp genome, with the search parameters set at ≥10 repeat units for mononucleotides, ≥8 repeat units for dinucleotides, ≥4 repeat units for trinucleotides and tetranucleotides, and ≥3 repeat units for pentanucleotide and hexanucleotide SSRs. Tandem repeats in the *A*. *lyrata* and *A*. *halleri* cp genomes were identified using Tandem Repeats Finder version 4.07 b with default settings^96^.

### Sequence Divergence and Phylogenetic Analysis

Complete cp genomes and a separate partition containing only the 70 shared genes were used to analyse the average pairwise sequence divergence for ten *Arabidopsis* species: *A*. *thaliana*, *A*. *arenosa*, *A*. *cebennensis*, *A*. *pedemontana*, *A*. *arenicola*, *A*. *croatica*, *A*. *neglecta*, *A*. *petrogena*, *A*. *suecica* and *A*. *umezawana*. Missing and ambiguous gene annotations were confirmed by comparative sequence analysis after a multiple sequence alignment and gene order comparison. These regions were aligned using MAFFT (version 7.222)^97^ with default parameters. Kimura’s two parameter (K2P) model was selected to calculate pairwise sequence divergences^98^. To resolve the *A*. *halleri* ssp. *gemmifera* and *A*. *lyrata* ssp. *petraea* phylogenetic positions within the family *Brassicaceae*, twenty-nine published cp genomes were downloaded from the NCBI database for analysis. First, multiple alignments were performed using complete cp genomes based on the conserved structure and gene order of chloroplast genomes^98^. Four methods were employed to construct the phylogenetic trees, including Bayesian inference (BI) implemented with MrBayes 3.1.2^99^, maximum parsimony (MP) with PAUP 4.0^100^, maximum likelihood (ML) and neighbour-joining (NJ) with MEGA 6^92^ using settings derived from Wu *et al*.^101^. MP was run using a heuristic search with 1000 random addition sequence replicates with the tree-bisection-reconnection (TBR) branch-swapping tree search criterion. Parameters for the ML analysis were optimized with a BIONJ tree as the starting tree with 1000 bootstrap replicates using the Kimura 2-parameter model with gamma-distributed rate heterogeneity and invariant sites. For Bayesian posterior probabilities (PP) in the BI analyses, the best substitution model GTR + G model was tested according to the Akaike information criterion (AIC) by jModelTest verion 2^102^. The Markov Chain Monto Carlo (MCMC) was run for 1,000,000 generations with 4 incrementally heated chains, starting from random trees and sampling 1 out of every 100 generations. The first 25% of trees were discarded as burn-in to estimate the value of posterior probabilities. In the second phylogenetic analysis, 70 shared genes from the cp genomes of the twenty-two *Brassicaceae* members, with *Carica papaya* as the outgroup species, were aligned in ClustalX using the default settings, followed by manual adjustment to preserve reading frames. The above four phylogenetic-inference methods were used to infer trees from these 65 concatenated genes using the same settings described above and in Yao *et al*.^103^.

## Electronic supplementary material

SI All files

SI Table 9
