# Supplementary material for: Chloroplast genomes of Arabidopsis halleri ssp. gemmifera and Arabidopsis lyrata ssp. petraea: Structures and comparative analysis
Source: Sci Rep. 2017 Aug 8;7:7556. doi: 10.1038/s41598-017-07891-5 (PMC5548756; doi:10.1038/s41598-017-07891-5)
Supplement: Supplementary file 1 — SI All files [file 41598_2017_7891_MOESM1_ESM.docx]

**Chloroplast genomes of** ***Arabidopsis halleri* ssp. *gemmifera* and *Arabidopsis lyrata* ssp. *petraea:* Structures and comparative analysis**

Sajjad Asaf^1^, Abdul Latif Khan^2^, Muhammad Aaqil Khan^1^, Muhammad Waqas^1^, Sang-Mo Kang^1^, Byung-Wook Yun^1^, In-Jung Lee^1^*

*1School of Applied Biosciences, Kyungpook National University, Daegu 41566, Republic of Korea*

*2Chair of Oman's Medicinal Plants & Marine Natural Products, University of Nizwa, Nizwa 616, Oman*

Correspondence: In-Jung Lee, Crop Physiology Laboratory, School of Applied Biosciences, Kyungpook National University, Daegu 41566, Republic of Korea. Tel: + 82-53-950-5708. E-mail: [ijlee@knu.ac.kr](mailto:ijlee@knu.ac.kr)

**Supporting Information**

**Table S1.** Primers used for gap closure and assembly validation in *A. halleri* ssp. *gemmifera* and *A. lyrata* ssp. *petraea*

**Table S2.** Primers used to confirm the junctions of the inverted repeats (IR) regions in *A. halleri* ssp. *gemmifera* and *A. lyrata* ssp. *petraea.*

**Table S3**. The codon–anticodon recognition pattern and codon usage for the *A. halleri* ssp. *gemmifera* chloroplast genome**.**

**Table S4**. The codon–anticodon recognition pattern and codon usage for the *A. lyrata* ssp. *petraea* chloroplast genome.

**Table S5.** Repeat sequences in the *A. halleri* ssp. *gemmifera* chloroplast genome*.*

**Table S6.** Repeat sequences in the *A. lyrata* ssp. *petraea* chloroplast genome*.*

**Table S7.** Simple sequence repeats (SSRs) in the *A. halleri* ssp. *gemmifera* chloroplast genome*.*

**Table S8.** Simple sequence repeats (SSRs) in the *A. lyrata* ssp. *petraea* chloroplast genome*.*

**Table S9.** Average pairwise sequence distance of A. halleri ssp. gemmifera and A. lyrata ssp. petraea with ten *arabidopsis* species cp genomes.

**S10 Table.** The genes with introns in the *A. halleri* ssp. *gemmifera* chloroplast genome and the length of exons and introns.

**S11 Table**. The genes with introns in the *A. lyrata* ssp. *petraea* chloroplast genome and the length of exons and introns.

**Table S1. Primers used for gap closure and assembly validation in** ***A. halleri* ssp. *gemmifera* and *A. lyrata* ssp. *petraea***

| Number | Primers | Sequence | Size | cp genome |
| --- | --- | --- | --- | --- |
| 1 | 30588AB-F | TTGTCAATATAATGAAATGGTATG | 574 bp | *A. halleri* ssp. *gemmifera* |
|  | 31132AB-R | AAATTGCCTAGCGAAATCGAACG |  |  |
| 2 | 62212BC-F | GGAAGGGGTCAGATTTATCCTGAT | 229bp | *A. halleri* ssp. *gemmifera* |
|  | 62440BC-R | GATTACTCGTTAATGGTTGATCGA |  |  |
| 3 | 80918CD-F | CTTCTGATCTTTCTAGAGGAGTAT | 614bp | *A. halleri* ssp. *gemmifera* |
|  | 81531CD-R | GGCAGATATGCTCTTCAAACACTT |  |  |
| 4 | 100972EF-F | CTTGGAATGAAAGACAATTCCGAA | 296bp | *A. halleri* ssp. *gemmifera* |
|  | 101288EF-R | CTTGGTAAGCTATTGCCTCACCAA |  |  |
| 5 | 131955GH-F | GTTACGCCTTTCGTGCGGGTCGGA | 648bp | *A. halleri* ssp. *gemmifera* |
|  | 132535GH-R | CGTAGTCGATGGACAACAGGTGAA |  |  |
| 6 | 154435IJ-F | GGCGGAAAAAGAAATCATAATAAC | 490bp | *A. halleri* ssp. *gemmifera* |
|  | 433IJ-R | AGCTGCTGTTGAGGCTCCATCTAC |  |  |
| 7 | 31635KL-F | GATAAGTCGTCTCTTGAATCG | 849bp | *A. lyrata* ssp. *petraea* |
|  | 31502KL-R | CGCATCAACTCAATTTGTTTT |  |  |
| 8 | 56366MN-F | GTCGATAAATTAGATGGCCAAG | 505bp | *A. lyrata* ssp. *petraea* |
|  | 56870MN-R | CTTATATTGTGTAAAATTCTC |  |  |
| 9 | 79559OP-F | CAAATTTTACGAACGGAAGCC | 932bp | *A. lyrata* ssp. *petraea* |
|  | 80518OP-R | AAAGAACATGTTGATTATATC |  |  |
| 10 | 128307QR-F | GTTTTATAGTTATAGTATGTTCGA | 550bp | *A. lyrata* ssp. *petraea* |
|  | 128920QR-R | AGTGTTTGTGATTGTTCCAGAAG |  |  |

**Table S2. Primers used to confirm the junctions of the inverted repeats (IR) regions in *A. halleri* ssp. *gemmifera* and *A. lyrata* ssp. *petraea.***

|  | *Arabidopsis halleri* | | Size |
| --- | --- | --- | --- |
| 1 | 83888-F | ACTCTCCGTGCTTTATGGGC | 739 |
|  | 84626-R | CAACAGTCGGACAAGTGGGA |  |
| 2 | 110272-F | TCGGAAGAAGGGGAAGATCT | 255 |
|  | 110527-R | GGTGGAAGTCGCATCTCTTC |  |
| 3 | 132912-F | TAGGGGCCTTAGCTGGTGAT | 824 |
|  | 133736-R | AAGCGATTCCCGTAGTAGCG |  |
| 4 | 152283-F | CGCCCAACTCATAATTGGCG | 173 |
|  | 152456-R | CGCCAATTATGAGTTGGGCG |  |
|  | *Arabidopsis lyrata* | |  |
| 5 | 84159-F | GATGCCCGGGACCAAGTTAT | 266 |
|  | 84425-R | AAAAACCCGTAACCCCCTGG |  |
| 6 | 110249-F | GAAAATCTAGATGGAAATA | 356 |
|  | 110605-R | CTTTTTTTAAAACATCTTTA |  |
| 7 | 128190-F | TCGAAAACTAGAGTTACAAATG | 146 |
|  | 128336-R | CCACGTTCGAACATACTATAAC |  |
| 8 | 154455-F | GAAGCTTAATACAAAGGCGG | 120 |
|  | 29-R | CGCGGGTTCAATTCCCGTCG |  |

**Table S3**. **The codon–anticodon recognition pattern and codon usage for the *A. halleri* ssp. *gemmifera* chloroplast genome.**

| **Amino acid** | **Codon** | **No** | **RSCU** | **tRNA** | **Amino acid** | **Codon** | **No** | **RSCU** | **tRNA** |
| --- | --- | --- | --- | --- | --- | --- | --- | --- | --- |
| Phe | UUU | 997 | 1.33 |  | Ala | GCA | 371 | 1.10 | *trnA-UGC* |
| Phe | UUC | 502 | 0.66 | *trnF-GAA* | Ala | GCG | 137 | 0.40 |  |
| Leu | UUA | 898 | 2.01 | *trnL-UAA tRNA* | Tyr | UAU | 755 | 1.63 |  |
| Leu | UUG | 509 | 1.14 | *trnL-CAA tRNA* | Tyr | UAC | 167 | 0.36 | *trnY-GUA tRNA* |
| Leu | CUU | 563 | 1.26 |  | Stop | UAG | 23 | 0.80 |  |
| Leu | CUC | 181 | 0.40 |  | Stop | UGA | 10 | 0.l34 |  |
| Leu | CUA | 358 | 0.80 | *trnL-UAG tRNA* | Stop | UAA | 53 | 1.84 |  |
| Leu | CUG | 166 | 0.37 |  | His | CAU | 443 | 1.51 |  |
| Ile | AUU | 1077 | 1.49 |  | His | CAC | 142 | 0.48 | *trnH-GUG tRNA* |
| Ile | AUC | 407 | 0.56 | *trnI-GAU tRNA* | Gln | CAA | 698 | 1.56 | *trnQ-UUG tRNA* |
| Ile | AUA | 671 | 0.93 |  | Gln | CAG | 194 | 0.43 |  |
| Met | AUG | 576 | 1 | *trnM-CAU tRNA* | Asn | AAU | 899 | 1.53 |  |
| Val | GUU | 505 | 1.49 |  | Asn | AAC | 273 | 0.46 | *trnQ-UUG tRNA* |
| Val | GUC | 169 | 0.49 | *trnV-GAC tRNA* | Lys | AAA | 984 | 1.51 | *trnK-UUU tRNA* |
| Val | GUA | 480 | 1.41 | *trnV-UAC tRNA* | Lys | AAG | 319 | 0.48 |  |
| Val | GUG | 200 | 0.59 |  | Asp | GAU | 805 | 1.62 |  |
| Ser | UCU | 548 | 1.70 |  | Asp | GAC | 186 | 0.37 | *trnD-GUC tRNA* |
| Ser | UCC | 292 | 0.90 | *trnS-GGA tRNA* | Glu | GAA | 951 | 1.49 | *trnE-UUC tRNA* |
| Ser | UCA | 388 | 1.20 | *trnS-UGA tRNA* | Glu | GAG | 318 | 0.50 |  |
| Ser | UCG | 190 | 0.58 |  | Cys | UGU | 229 | 1.48 |  |
| Ser | AGU | 390 | 1.21 |  | Cys | UGC | 79 | 0.51 |  |
| Ser | AGC | 125 | 0.38 | *trnS-GCU tRNA* | Trp | UGG | 423 | 1 | *trnW-CCA tRNA* |
| Pro | CCU | 411 | 1.61 |  | Arg | CGU | 321 | 1.30 | *trnR-ACG tRNA* |
| Pro | CCC | 186 | 0.73 |  | Arg | CGC | 112 | 0.45 |  |
| Pro | CCA | 288 | 1.13 | *trnP-UGG tRNA* | Arg | CGA | 352 | 1.42 |  |
| Pro | CCG | 133 | 0.52 |  | Arg | CGG | 113 | 0.45 |  |
| Thr | ACU | 519 | 1.61 |  | Arg | AGA | 424 | 1.71 | *trnR-UCU tRNA* |
| Thr | ACC | 230 | 0.71 | *trnT-GGU tRNA* | Arg | AGG | 159 | 0.64 |  |
| Thr | ACA | 396 | 1.23 | *trnT-UGU tRNA* | Gly | GGU | 568 | 1.31 |  |
| Thr | ACG | 137 | 0.42 |  | Gly | GGC | 160 | 0.37 |  |
| Ala | GCU | 632 | 1.87 |  | Gly | GGA | 712 | 1.65 | *trnG-UCC tRNA* |
| Ala | GCC | 209 | 0.61 |  | Gly | GGG | 284 | 0.65 |  |

**Table S4**. **The codon–anticodon recognition pattern and codon usage for the *A. lyrata* ssp. *petraea* chloroplast genome.**

| **Amino acid** | **Codon** | **No** | **RSCU** | **tRNA** | **Amino acid** | **Codon** | **No** | **RSCU** | **tRNA** |
| --- | --- | --- | --- | --- | --- | --- | --- | --- | --- |
| Phe | UUU | 996 | 1.32 |  | Ala | GCA | 371 | 1.09 | *trnA-UGC* |
| Phe | UUC | 503 | 0.67 | *trnF-GAA* | Ala | GCG | 137 | 0.40 |  |
| Leu | UUA | 894 | 2.01 | *trnL-UAA tRNA* | Tyr | UAU | 759 | 1.63 |  |
| Leu | UUG | 510 | 1.14 | *trnL-CAA tRNA* | Tyr | UAC | 167 | 0.36 | *trnY-GUA tRNA* |
| Leu | CUU | 563 | 1.26 |  | Stop | UAG | 21 | 0.73 |  |
| Leu | CUC | 182 | 0.40 |  | Stop | UGA | 13 | 0.45 |  |
| Leu | CUA | 355 | 0.79 | *trnL-UAG tRNA* | Stop | UAA | 52 | 1.81 |  |
| Leu | CUG | 164 | 0.36 |  | His | CAU | 440 | 1.51 |  |
| Ile | AUU | 1077 | 1.50 |  | His | CAC | 142 | 0.48 | *trnH-GUG tRNA* |
| Ile | AUC | 406 | 0.56 | *trnI-GAU tRNA* | Gln | CAA | 698 | 1.56 | *trnQ-UUG tRNA* |
| Ile | AUA | 667 | 0.93 |  | Gln | CAG | 196 | 0.43 |  |
| Met | AUG | 574 | 1 | *trnM-CAU tRNA* | Asn | AAU | 897 | 1.53 |  |
| Val | GUU | 506 | 1.49 |  | Asn | AAC | 274 | 0.46 | *trnQ-UUG tRNA* |
| Val | GUC | 168 | 0.49 | *trnV-GAC tRNA* | Lys | AAA | 984 | 1.51 | *trnK-UUU tRNA* |
| Val | GUA | 482 | 1.42 | *trnV-UAC tRNA* | Lys | AAG | 317 | 0.48 |  |
| Val | GUG | 199 | 0.58 |  | Asp | GAU | 802 | 1.62 |  |
| Ser | UCU | 545 | 1.69 |  | Asp | GAC | 186 | 0.37 | *trnD-GUC tRNA* |
| Ser | UCC | 294 | 0.91 | *trnS-GGA tRNA* | Glu | GAA | 952 | 1.49 | *trnE-UUC tRNA* |
| Ser | UCA | 388 | 1.20 | *trnS-UGA tRNA* | Glu | GAG | 318 | 0.50 |  |
| Ser | UCG | 192 | 0.59 |  | Cys | UGU | 230 | 1.49 |  |
| Ser | AGU | 390 | 1.21 |  | Cys | UGC | 78 | 0.50 |  |
| Ser | AGC | 124 | 0.38 | *trnS-GCU tRNA* | Trp | UGG | 424 | 1 | *trnW-CCA tRNA* |
| Pro | CCU | 412 | 1.62 |  | Arg | CGU | 318 | 1.28 | *trnR-ACG tRNA* |
| Pro | CCC | 186 | 0.73 |  | Arg | CGC | 113 | 0.45 |  |
| Pro | CCA | 287 | 1.12 | *trnP-UGG tRNA* | Arg | CGA | 352 | 1.42 |  |
| Pro | CCG | 132 | 0.51 |  | Arg | CGG | 113 | 0.45 |  |
| Thr | ACU | 521 | 1.62 |  | Arg | AGA | 424 | 1.71 | *trnR-UCU tRNA* |
| Thr | ACC | 229 | 0.71 | *trnT-GGU tRNA* | Arg | AGG | 161 | 0.65 |  |
| Thr | ACA | 396 | 1.23 | *trnT-UGU tRNA* | Gly | GGU | 567 | 1.31 |  |
| Thr | ACG | 135 | 0.42 |  | Gly | GGC | 160 | 0.37 |  |
| Ala | GCU | 631 | 1.86 |  | Gly | GGA | 715 | 1.65 | *trnG-UCC tRNA* |
| Ala | GCC | 211 | 0.62 |  | Gly | GGG | 285 | 0.66 |  |

**Table S5. Repeat sequences in the *A. halleri* ssp. *gemmifera* chloroplast genome*.***

| **Repeat type** | **Length** | **Position A** | **Locus** | **Position B** | **Locus** |
| --- | --- | --- | --- | --- | --- |
| F | 99 | 47937 | IGS | 48048 | IGS |
| F | 67 | 38734 | *psaB* (CDS) | 40958 | *psaA* (CDS) |
| F | 52 | 38755 | *psaB* (CDS) | 40979 | *psaA* (CDS) |
| F | 45 | 89085 | *ycf2* (CDS) | 89109 | *ycf2* (CDS) |
| F | 45 | 149516 | *ycf2* (CDS) | 149540 | *ycf2* (CDS) |
| P | 45 | 89085 | *ycf2* (CDS) | 149516 | *ycf2* (CDS) |
| P | 45 | 89109 | *ycf2* (CDS) | 149540 | *ycf2* (CDS) |
| F | 44 | 47737 | IGS( *trnL-UAA, trnF-GAA*) | 47945 | IGS( *trnL-UAA*, *trnF-GAA)* |
| F | 44 | 47737 | IGS( *trnL-UAA, trnF-GAA*) | 48056 | IGS( *trnL-UAA, trnF-GAA*) |
| F | 43 | 113 | IGS (*trnH-GUG, psbA*) | 114 | IGS (*trnH-GUG, psbA*) |
| P | 40 | 120399 | IGS *ndhA* (intron) | 139767 | IGS (*trnV-GAC*, *rps12*) |
| F | 40 | 98863 | IGS( *rps12, trnV-GAC*) | 120399 | IGS *ndhA* (intron) |
| F | 39 | 43829 | *ycf3*( intron) | 98863 | IGS( *rps12, trnV-GAC*) |
| F | 39 | 47783 | IGS( *trnL-UAA, trnF-GAA*) | 47892 | IGS( *trnL-UAA, trnF-GAA)* |
| P | 39 | 43829 | *ycf3* ( intron) | 139768 | IGS (*trnV-GAC*, *rps12*) |
| F | 38 | 113705 | IGS (*rpl32, trnL-UAG*) | 113724 | IGS (*rpl32, trnL-UAG*) |
| F | 35 | 47783 | IGS ( *trnL-UAA, trnF-GAA*) | 47991 | IGS( *trnL-UAA, trnF-GAA*) |
| F | 35 | 47783 | IGS ( *trnL-UAA, trnF-GAA*) | 48102 | IGS( *trnL-UAA, trnF-GAA*) |
| F | 34 | 47859 | IGS ( *trnL-UAA, trnF-GAA*) | 47891 | IGS (*trnL-UAA, trnF-GAA*) |
| F | 34 | 47839 | IGS ( *trnL-UAA, trnF-GAA*) | 47970 | IGS( *trnL-UAA, trnF-GAA*) |
| P | 33 | 89097 | *ycf2* (CDS) | 149516 | *ycf2* (CDS) |
| P | 33 | 89121 | *ycf2* (CDS) | 149540 | *ycf2* (CDS) |
| P | 33 | 107784 | IGS (RNA-RNA) | 130821 | IGS (RNA-RNA) |
| P | 33 | 107816 | IGS (RNA-RNA) | 130853 | IGS (RNA-RNA) |
| F | 33 | 89097 | *ycf2* (CDS) | 89121 | *ycf2* (CDS) |
| F | 33 | 107784 | IGS (RNA-RNA) | 107816 | IGS (RNA-RNA) |
| F | 33 | 130821 | IGS (RNA-RNA) | 130853 | IGS (RNA-RNA) |
| P | 32 | 89131 | *ycf2* (CDS) | 149486 | *ycf2* (CDS) |
| P | 32 | 89152 | *ycf2* (CDS) | 149507 | *ycf2* (CDS) |
| F | 32 | 47749 | IGS ( *trnL-UAA, trnF-GAA*) | 47826 | IGS (*trnL-UAA, trnF-GAA*) |
| F | 32 | 47850 | IGS ( *trnL-UAA, trnF-GAA*) | 47981 | IGS(*trnL-UAA, trnF-GAA*) |
| F | 32 | 47850 | IGS ( *trnL-UAA, trnF-GAA*) | 48092 | IGS (*trnL-UAA, trnF-GAA*) |
| F | 32 | 89131 | *ycf2* (CDS) | 89152 | *ycf2* (CDS) |
| F | 32 | 149486 | *ycf2* (CDS) | 149507 | *ycf2* (CDS) |
| F | 30 | 36270 | IGS (*lhbA, trnG-UCC*) | 36319 | IGS (*lhbA-trnG-UCC*) |
| F | 30 | 112 | IGS (*trnH-GUG, psbA*) | 114 | IGS (*trnH-GUG,psbA*) |
| F | 30 | 77859 | IGS (*petD, rpoA*) | 77888 | IGS (*petD-rpoA*) |
| F | 30 | 149531 | *ycf2* (CDS) | 149555 | *ycf2* (CDS) |

**Table S6. Repeat sequences in the *A. lyrata* ssp. *petraea* chloroplast genome*.***

| **Repeat type** | **Length (bp)** | **Position A** | **Locus** | **Position B** | **Locus** |
| --- | --- | --- | --- | --- | --- |
| **F** | 101 | 47678 | IGS (*trnL-UAA, trnF-GAA*) | 47878 | IGS (*trnL-UAA, trnF-GAA*) |
| **F** | 67 | 38715 | *psaB* (CDS) | 40939 | *psaA* (CDS) |
| **F** | 52 | 38736 | *psaB* (CDS) | 40960 | *psaA* (CDS) |
| **F** | 50 | 7183 | IGS (*psbK, psbI*) | 7233 | IGS (*psbK, psbI*) |
| **P** | 45 | 89046 | *ycf2* (CDS) | 149556 | *ycf2* (CDS) |
| **P** | 45 | 89070 | *ycf2* (CDS) | 149556 | *ycf2* (CDS) |
| **F** | 45 | 89046 | *ycf2* (CDS) | 89070 | *ycf2* (CDS) |
| **F** | 45 | 149532 | *ycf2* (CDS) | 149556 | *ycf2* (CDS) |
| **F** | 44 | 8096 | IGS (*trnS-GCU,trnR-UCU*) | 8172 | IGS (*trnS-GCU,trnR-UCU*) |
| **F** | 41 | 47719 | IGS (*trnL-UAA, trnF-GAA*) | 48090 | IGS (*trnL-UAA, trnF-GAA*) |
| **F** | 40 | 98821 | IGS (*rps12, trnV-GAC*) | 120422 | *ndhA* (intron) |
| **P** | 40 | 120422 | *ndhA* (intron) | 139786 | IGS (*trnV-GAC, rps12*) |
| **F** | 39 | 43794 | *ycf3* ( intron) | 98821 | IGS (*rps12, trnV-GAC*) |
| **P** | 39 | 43794 | *ycf3* ( intron) | 139787 | IGS (*trnV-GAC, rps12*) |
| **F** | 38 | 47706 | IGS (*trnL-UAA, trnF-GAA*) | 47782 | IGS (*trnL-UAA, trnF-GAA*) |
| **F** | 38 | 47782 | IGS (*trnL-UAA, trnF-GAA*) | 47906 | IGS (*trnL-UAA, trnF-GAA*) |
| **F** | 37 | 47728 | IGS (*trnL-UAA, trnF-GAA*) | 48099 | IGS (*trnL-UAA, trnF-GAA*) |
| **F** | 37 | 47784 | IGS (*trnL-UAA, trnF-GAA*) | 47975 | IGS (*trnL-UAA, trnF-GAA*) |
| **F** | 37 | 47928 | IGS (*trnL-UAA, trnF-GAA*) | 48099 | IGS (*trnL-UAA, trnF-GAA*) |
| **F** | 36 | 77812 | IGS (*petD, rpoA*) | 77843 | IGS (*petD, rpoA*) |
| **F** | 33 | 47795 | IGS (*trnL-UAA, trnF-GAA*) | 47986 | IGS (*trnL-UAA, trnF-GAA*) |
| **P** | 33 | 89058 | *ycf2* (CDS) | 149532 | *ycf2* (CDS) |
| **P** | 33 | 89082 | *ycf2* (CDS) | 149556 | *ycf2* (CDS) |
| **P** | 33 | 107736 | IGS (RNA, RNA) | 130846 | IGS (RNA, RNA) |
| **P** | 33 | 107768 | IGS (RNA, RNA) | 130878 | IGS (RNA, RNA) |
| **F** | 33 | 89058 | *ycf2* (CDS) | 89082 | *ycf2* (CDS) |
| **F** | 33 | 107736 | IGS (RNA, RNA) | 107768 | IGS (RNA, RNA) |
| **F** | 33 | 130846 | IGS (RNA, RNA) | 130878 | IGS (RNA, RNA) |
| **P** | 32 | 89092 | *ycf2* (CDS) | 149502 | *ycf2* (CDS) |
| **P** | 32 | 89113 | *ycf2* (CDS) | 149523 | *ycf2* (CDS) |
| **F** | 32 | 8108 | IGS (*trnS-GCU, trnR-UCU*) | 8184 | IGS (*trnS-GCU, trnR-UCU*) |
| **F** | 32 | 89092 | *ycf2* (CDS) | 89113 | *ycf2* (CDS) |
| **F** | 32 | 149502 | *ycf2* (CDS) | 149523 | *ycf2* (CDS) |
| **F** | 31 | 47694 | IGS (*trnL-UAA, trnF-GAA*) | 48053 | IGS (*trnL-UAA, trnF-GAA*) |
| **F** | 31 | 47894 | IGS (*trnL-UAA, trnF-GAA*) | 48053 | IGS (*trnL-UAA, trnF-GAA*) |
| **P** | 30 | 7793 | *trnS-GCU* | 44889 | *trnS-GCU* |
| **P** | 30 | 43806 | *ycf3* ( intron) | 139784 | IGS (*trnV-GAC, rps12*) |
| **F** | 30 | 149547 | *ycf2* (CDS) | 149571 | *ycf2* (CDS) |
| **F** | 30 | 43806 | *ycf3* ( intron) | 98833 | IGS ( *rps12, trnV-GAC*) |
| **F** | 30 | 47714 | IGS (*trnL-UAA, trnF-GAA*) | 47790 | IGS (*trnL-UAA, trnF-GAA*) |
| **F** | 30 | 47790 | IGS (*trnL-UAA, trnF-GAA*) | 47914 | IGS (*trnL-UAA, trnF-GAA*) |
| **F** | 30 | 47820 | IGS (*trnL-UAA, trnF-GAA*) | 47852 | IGS (*trnL-UAA, trnF-GAA*) |

**Table S7.** Simple sequence repeats (SSRs) in the *A. halleri* ssp. *gemmifera* chloroplast genome*.*

| **Unit** | **Length** | **No** | **SSR start** |
| --- | --- | --- | --- |
| **A** | 19 | 1 | 49953 |
|  | 18 | 1 | 114 |
|  | 17 | 1 | 174 |
|  | 16 | 3 | 30083, 65403, 114998 |
|  | 15 | 9 | 199, 7553, 28851, 30870, 65289, 70397, 82226,99352, 139282 |
|  | 14 | 3 | 4118, 32381, 78083 |
|  | 13 | 6 | 4635, 7918, 31670, 42557, 68329, 124142 |
|  | 12 | 8 | 2307, 56836, 57372, 63145, 82589, 113273, 120797, 127099 |
|  | 11 | 11 | 1610, 17935, 22416, 28690,44448, 50842, 59689, 66634, 67147, 81910, 120995, 127069 |
|  | 10 | 32 | 1784, 4038, 12848, 13233, 14834, 22467, 25692, 26875, 27366, 29075, 30838, 31027, 36703, 42209, 50445, 51292, 54097, 56500, 65614, 70519, 71361, 82443, 100152, 108099, 110399, 115483, 124309,125830, 127039, 128240, 130540, 138487 |
| **C** | 11 | 1 | 67147 |
|  | 10 | 1 | 48525 |
| **AT** | 23 | 1 | 112682 |
|  | 15 | 2 | 8003, 8062 |
|  | 14 | 1 | 8206 |
|  | 13 | 2 | 13722, 67752 |
|  | 11 | 6 | 8130, 19307, 31318, 94504, 113330, 144134 |
|  | 10 | 7 | 3828, 6374, 8102, 8196, 8223, 36352, 63121, |
|  | 9 | 19 | 12654, 20347, 27358, 28161, 31306, 35325, 36363, 45992, 60788, 6 |
|  |  |  | 1706, 66856, 84872, 91774, 99205, 102716, 135924, 139435, 146866, 153768 |
|  | 8 | 37 |  |
| **AG** | 9 | 9 | 28161, 35325, 60788, 91774, 99205, 102716, 135924, 139435, 146866 |
|  | 8 | 11 | 86512, 87499, 89741, 95276, 106367, 111409, 132274, 143365,148900, |
|  |  |  | 151142, 152129 |
| **AC** | 9 | 1 | 12654 |
|  | 8 | 1 | 6444 |
| **AAT** | 14 | 1 | 46579 |
|  | 12 | 1 | 12983 |
|  | 11 | 2 | 28842, 57341 |
|  | 10 | 2 | 51246, 123634 |
|  | 9 | 11 | 4691, 4703, 4715, 8056, 15012, 31543, 31553, 80584,112050, 114631, 119162 |
| **AAG** | 12 | 1 | 44136 |
|  | 11 | 4 | 718, 111171, 127016, 127571 |
|  | 10 | 5 | 27838, 78174, 97015, 127550, 141624 |
|  | 9 | 14 | 37215, 66596, 83364, 84381, 90421, 90527, 92257, 95571, 120219, 143069,  146383, 148113, 148219, 154259 |
| **AATT** | 16 | 1 | 77767 |
|  | 12 | 1 | 66086 |
| **AAAG** | 16 | 1 | 82361 |
| **AAAT** | 15 | 1 | 4528 |
|  | 13 | 1 | 65108 |
|  | 12 | 2 | 46439, 63174 |
| **AGAT** | 13 | 2 | 46469, 112444 |
| **AAAC** | 12 | 1 | 28431 |
| **ATCC** | 12 | 2 | 93528, 145109 |
| **AAATAG** | 20 | 1 | 67826 |
| **AAATCT** | 19 | 1 | 126662 |
| **AATATT** | 18 | 2 | 94511, 144120 |

**Table S8. Simple sequence repeats (SSRs) in the *A. lyrata* ssp. *petraea* chloroplast genome***.*

| Unit | **Length** | **No** | **SSR start** |
| --- | --- | --- | --- |
| **A** | 31 | 1 | 144 |
|  | 17 | 2 | 99395, 139260 |
|  | 16 | 3 | 14820, 42587, 49944 |
|  | 15 | 1 | 32375 |
|  | 14 | 7 | 28673, 28840, 46450, 65305, 68345, 78114, 114957 |
|  | 13 | 10 | 7595, 30072, 31018, 44483, 52221, 63154, 65421, 70423, 113858, 124,113 |
|  | 12 | 10 | 2321, 4133, 7954, 8010, 8365, 12355, 57369, 81951, 113968, 127073, |
|  | 11 | 14 | 4053, 17921, 27349, 30865, 31666, 31708, 36721, , 56834, 66650, 99431, 127012, 127043, 139230 |
|  | 10 | 20 | 1798, 4539, 12821, 13206, 15824, 22454, 25679, 28268, 31163, 42228, 50436, 54091,56498, 59697, 60370, 70647, 71196, 71386, 82483, 100200, 108147, 110448, 115442, 120968, 124280, 125801, 128214, 130515, 138462 |
| **C** | 11 | 1 | 48515 |
| **AT** | 17 | 1 | 8043 |
|  | 15 | 1 | 67764 |
|  | 14 | 2 | 4714, 4735 |
|  | 13 | 2 | 13702, 31314 |
|  | 12 | 1 | 8100 |
|  | 11 | 5 | 8160, 19293, 94546, 112731,144115, |
|  | 10 | 2 | 3843, 6408 |
|  | 9 | 21 | 12627, 20334, 27341, 28143, 31302, 35320, 36371, 36381, 46029, 60796, 61714, 66870, 84911, 91816, 99247, 102764, 113283, 135899, 139416, 146847, 153752 |
|  | 8 | 37 | 6478, 8171, 9794, 12110, 26895, 29514, 31656, 46109, 46721, 59104, 59754, 63112, 66882, 80560, 83916, 86551, 87538, 89783, 93136, 95318, 106415, 108557, 111458, 112839, 113292, 118205, 118222, 121362, 124346, 125245, 130107, 132249, 143346, 145528, 148881, 151126, 152113 |
| **AAT** | 14 | 3 | 229, 28828, 46618 |
|  | 12 | 1 | 12956 |
|  | 11 | 1 | 57338 |
|  | 10 | 3 | 51236, 65413, 123606 |
|  | 9 | 12 | 242, 4703, 8086, 8094,14997, 26866, 31541, 3155180615,112099, 114590, 119130 |
| **AATT** | 16 | 1 | 77800 |
| **AAAG** | 16 | 1 | 82401 |
| **AGAT** | 13 | 1 | 112493 |
| **AAAT** | 13 | 1 | 65122 |
|  | 12 | 2 | 46476, 63184 |
| **AAAC** | 12 | 1 | 28414 |
| **ATCC** | 12 | 2 | 93570, 145090 |
| **AATAT** | 15 | 1 | 36291 |

**Table S9. Average pairwise sequence distance of *A*. *halleri* ssp. *gemmifera* and *A*. *lyrata* ssp. *petraea* with ten *arabidopsis* species cp genomes**.

This table is provided in the form of a single file.

**S10 Table. The genes with introns in the *A. halleri* ssp. *gemmifera* chloroplast genome and the length of exons and introns.**

| **Gene** | **Location** | **Exon I (bp)** | **Intron 1 (bp)** | **Exon II (bp)** | **Intron II (bp)** | **Exon III (bp)** |
| --- | --- | --- | --- | --- | --- | --- |
| *atpF* | LSC | 144 | 715 | 411 |  |  |
| clpP | LSC | 69 | 866 | 297 | 492 | 252 |
| *ndhA* | LSC | 552 | 1089 | 531 |  |  |
| *ndhB*** | IRa | 777 | 686 | 756 |  |  |
| petB | LSC | 6 | 799 | 645 |  |  |
| petD | LSC | 8 | 708 | 475 |  |  |
| *rpl2*** | IRb | 393 | 680 | 435 |  |  |
| rpl16 | LSC | 9 | 1061 | 399 |  |  |
| rpoC1 | LSC | 447 | 768 | 1620 |  |  |
| rps12* |  | 114 | - | 232 | 543 | 26 |
| rps16 | LSC | 40 | 925 | 224 |  |  |
| *ycf3* | LSC | 129 | 718 | 228 | 790 | 153 |
| trnI-GAU ** | IRA | 42 | 724 | 35 |  |  |
| trnA-UGC** | IRA | 38 | 801 | 35 |  |  |
| trnV-UAC | LSC | 37 | 597 | 39 |  |  |
| trnL-UAA | LSC | 37 | 512 | 50 |  |  |
| trnG-UCC | LSC | 23 | 715 | 49 |  |  |
| trnK-UUU | LSC | 37 | 2561 | 35 |  |  |

*the rps12 gene is divided into 5`-rps12 in the LSC region and 3`-rps12 in the IR region.

** Duplicated genes

**S11 Table**. **The genes with introns in the *A. lyrata* ssp. *petraea* chloroplast genome and the length of exons and introns.**

| **Gene** | **Location** | **Exon I (bp)** | **Intron 1 (bp)** | **Exon II (bp)** | **Intron II (bp)** | **Exon III (bp)** |
| --- | --- | --- | --- | --- | --- | --- |
| *atpF* | LSC | 144 | 720 | 411 |  |  |
| clpP | LSC | 69 | 869 | 297 | 492 | 252 |
| *ndhA* | LSC | 552 | 1084 | 531 |  |  |
| *ndhB*** | IRb | 777 | 686 | 756 |  |  |
| petB | LSC | 6 | 800 | 645 |  |  |
| petD | LSC | 8 | 747 | 475 |  |  |
| *rpl2*** | IRb | 393 | 680 | 435 |  |  |
| rpl16 | LSC | 9 | 1053 | 399 |  |  |
| *rpoC1* | LSC | 447 | 768 | 1620 |  |  |
| rps12* |  | 114 | - | 26 | 537 | 232 |
| rps16 | LSC | 40 | 888 | 227 |  |  |
| *ycf3* | LSC | 129 | 718 | 228 | 790 | 153 |
| trnI-GAU ** | IRA | 35 | 724 | 42 |  |  |
| trnA-UGC** | IRA | 35 | 801 | 38 |  |  |
| trnV-UAC | LSC | 37 | 597 | 39 |  |  |
| trnL-UAA | LSC | 37 | 504 | 50 |  |  |
| trnG-UCC | LSC | 23 | 714 | 49 |  |  |
| trnK-UUU | LSC | 37 | 2562 | 35 |  |  |

*the rps12 gene is divided into 5`-rps12 in the LSC region and 3`-rps12 in the IR region.

** Duplicated genes


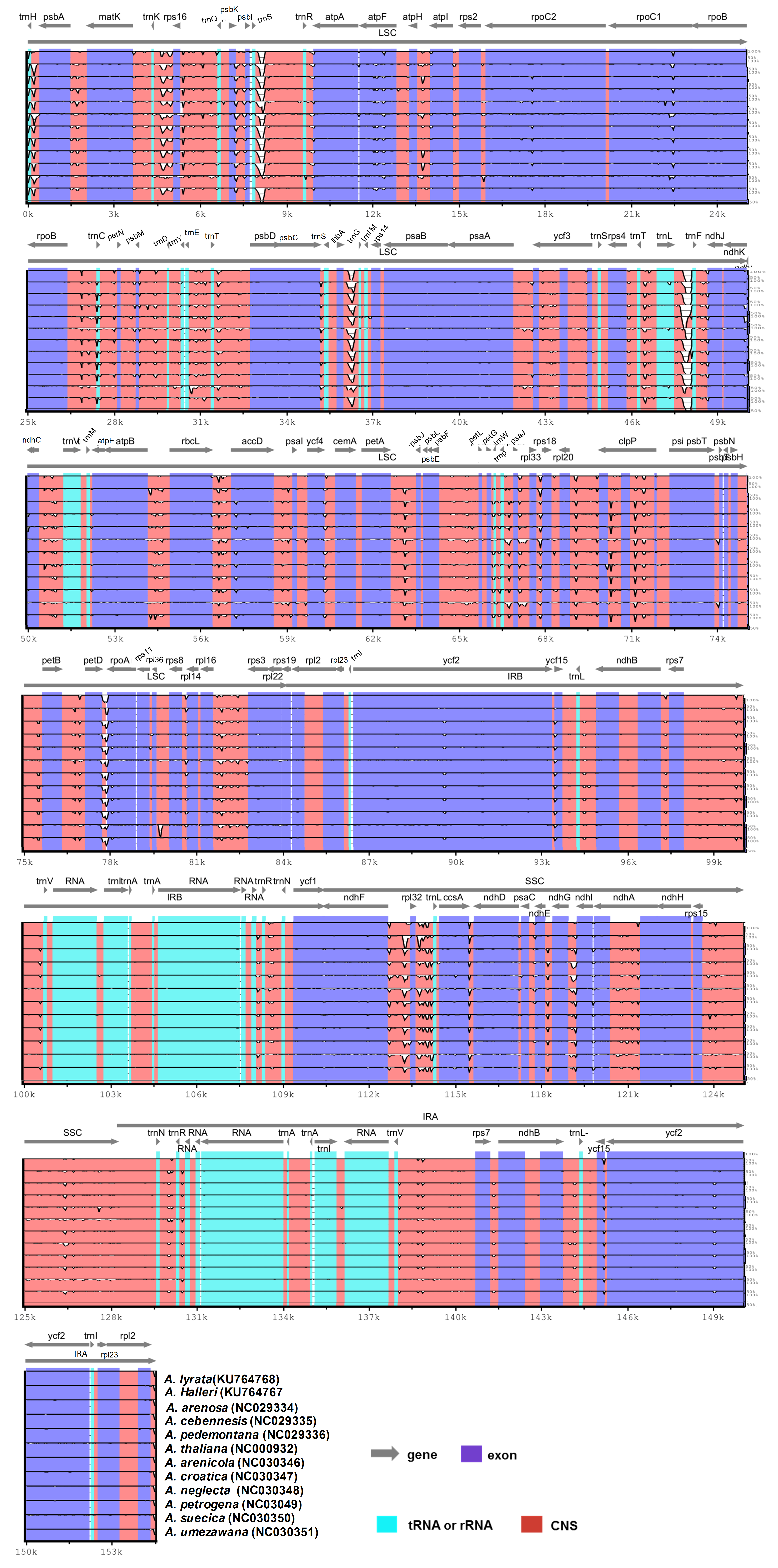


**Fig S1.** Alignment visualization of the twelve *Arabidopsis* chloroplast genome sequences. VISTA-based identity plot showing sequence identity among the six species using *A. lyrata* ssp. *petraea* as a reference. Vertical scale indicates the percentage of identity, ranging from 50% to 100%. Horizontal axis indicates the coordinates within the chloroplast genome. Arrows indicate the annotated genes and their transcriptional direction. The thick black lines show the inverted repeats (IRs) in the chloroplast genomes.





**Fig S2.** Pairwise sequence distance of *A. halleri* ssp. *gemmifera* genes with *A. lyrata* ssp. *petraea,* *A. thaliana*, *A. arenosa*, *A. cebennensis, A. pedemontana*, *A*. *arenicola*, *A*. *croatica*, *A*. *neglecta*, *A*. *petrogena*, *A*. *suecica* and *A*. *umezawana.*





**Fig S3.** Pairwise sequence distance of *A. lyrata* ssp. *petraea* genes with *A. halleri* ssp. *gemmifera*, *A. thaliana*, *A. arenosa*, *A. cebennensis, A. pedemontana*, *A*. *arenicola*, *A*. *croatica*, *A*. *neglecta*, *A*. *petrogena*, *A*. *suecica* and *A*. *umezawana.*


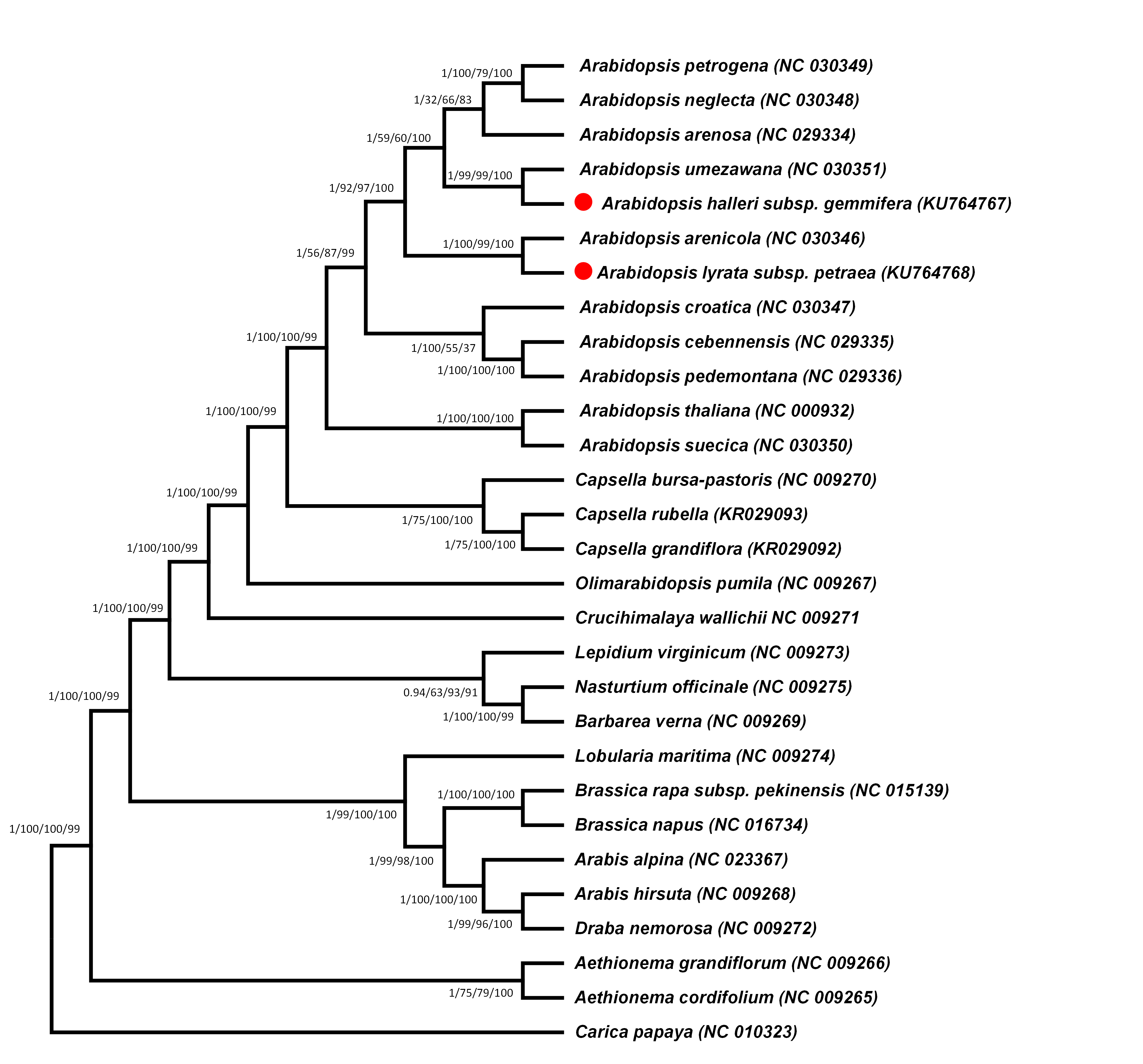


**Fig S4.** Phylogenetic trees were constructed for twenty-eight species from family Brassicaceae using different methods, and tree is shown for the whole genome sequence data sets. The whole genome sequence data set was used with four different methods, Bayesian inference (BI), maximum parsimony (MP), maximum likelihood (ML) and neighbor-joining (NJ). Numbers above the branches are the posterior probabilities of BI and bootstrap values of NJ, MP and ML respectively. Red dots represent the position for *A. halleri* ssp. *gemmifera* and *A. lyrata* ssp. *petraea*
